# Reproductive Biology and Its Impact on Body Size: Comparative Analysis of Mammalian, Avian and Dinosaurian Reproduction

**DOI:** 10.1371/journal.pone.0028442

**Published:** 2011-12-14

**Authors:** Jan Werner, Eva Maria Griebeler

**Affiliations:** Department of Ecology, Zoological Institute, University of Mainz, Mainz, Germany; Raymond M. Alf Museum of Paleontology, United States of America

## Abstract

Janis and Carrano (1992) suggested that large dinosaurs might have faced a lower risk of extinction under ecological changes than similar-sized mammals because large dinosaurs had a higher potential reproductive output than similar-sized mammals (JC hypothesis). First, we tested the assumption underlying the JC hypothesis. We therefore analysed the potential reproductive output (reflected in clutch/litter size and annual offspring number) of extant terrestrial mammals and birds (as “dinosaur analogs”) and of extinct dinosaurs. With the exception of rodents, the differences in the reproductive output of similar-sized birds and mammals proposed by Janis and Carrano (1992) existed even at the level of single orders. Fossil dinosaur clutches were larger than litters of similar-sized mammals, and dinosaur clutch sizes were comparable to those of similar-sized birds. Because the extinction risk of extant species often correlates with a low reproductive output, the latter difference suggests a lower risk of population extinction in dinosaurs than in mammals. Second, we present a very simple, mathematical model that demonstrates the advantage of a high reproductive output underlying the JC hypothesis. It predicts that a species with a high reproductive output that usually faces very high juvenile mortalities will benefit more strongly in terms of population size from reduced juvenile mortalities (e.g., resulting from a stochastic reduction in population size) than a species with a low reproductive output that usually comprises low juvenile mortalities. Based on our results, we suggest that reproductive strategy could have contributed to the evolution of the exceptional gigantism seen in dinosaurs that does not exist in extant terrestrial mammals. Large dinosaurs, e.g., the sauropods, may have easily sustained populations of very large-bodied species over evolutionary time.

## Introduction

Body size is one of the most fundamental attributes of any organism [1], [2]. While body size maxima for some organisms can be directly studied in living species, the largest terrestrial animals that have ever existed on earth, the sauropod dinosaurs, must be studied from the fossil record. Sander and Clauss [3] have argued that the gigantism of these animals must result from their unusual biology. Their thesis is supported by the observation that body size influences nearly every aspect of the biology of currently existing organisms and that many life history variables correlate with body size [4]–[6]. Variables such as mortality, age at sexual maturity, size or number of offspring are important for understanding life history strategies and population extinction risk, because such factors are directly related to the fitness of an organism [7]–[10]. These variables reflect several important trade-offs, e.g., investment of energy in somatic versus gonadic growth, in continuous or intermittent breeding, and in the investment in either many small or a few large offspring [11].

Kurtén [12] already pointed out that body size limits of a taxon reflect not only mechanical or physiological constrains, but also the scaling of its reproductive parameters [4], [11], [13]–[15]. Following the idea by Kurtén [12], Janis and Carrano (abbreviated hereafter as JC [16]) stated that terrestrial non-passerine birds, taken as a model for dinosaurs, differ from terrestrial mammals in terms of their reproductive biology. They found that, for terrestrial mammals, body mass was negatively correlated with litter size (number of offspring per litter; clutch size = number of offspring per clutch), breeding frequency (number of clutches/litters per year) and annual offspring number (total number of offspring per year = clutch/litter size×number of broods per year), whereas such relationships were absent in non-passerine birds.

Using terrestrial non-passerine birds as “dinosaur analogs”, JC put forward the hypothesis (henceforth called the JC hypothesis) that different reproductive strategies in dinosaurs and mammals (ovipary in birds and dinosaurs versus vivipary plus lactation in mammals) resulted in a different ability of dinosaurs and mammals to evolve and sustain large-bodied species over evolutionary time. JC suggested that, given their higher potential reproductive output (reflected in clutch size or number of offspring per year) compared to similar-sized mammals, large dinosaurs may have faced a lower risk of population extinction under ecological changes than mammals.

A higher potential reproductive output is advantageous when the size of a population is reduced, e.g., by a catastrophic event. While at equilibrium (at the carrying capacity of the population) mortalities are high in a population, because mortalities balance births; at lower population sizes mortalities are lower, and, thus, the number of surviving offspring per adult and reproduction event is much higher than at equilibrium [17], [18]. If population size is reduced and the species has a higher potential reproductive output, the species is able to increase population size faster than a species with a much lower potential reproductive output and a similar adult mortality. The faster a population reaches the equilibrium after a reduction in population size, the lower is its extinction risk and the higher is its chance to sustain viable populations over evolutionary time [19].

In the present study, we first tested the underlying assumptions of the JC hypothesis by comparing the potential reproductive output (reflected in clutch/litter size and annual offspring number) of terrestrial herbivorous mammals, birds and dinosaurs. However, our analysis differs profoundly from the original study: 1) we analysed a much larger sample size for all species groups (mammals, birds and dinosaurs); 2) we integrated, to our knowledge, the current information on body mass and reproduction of all dinosaur species for which this information is available at present; 3) we focused only on avian orders that are presumed to have less derived reproductive characteristics (e.g. ground breeding and precocial); 4) we restricted our analysis to avian and mammalian species with a similar diet (herbivorous) as assumed for the largest known dinosaurs, the sauropods [3], [20]; 5) we additionally controlled for phylogeny of species as body mass and reproduction characters are not statistically independent; and 6) we analysed single orders to test whether the dependencies between body mass and reproductive variables underlying the JC hypothesis exist within every avian and mammalian order.

Second, we present a very simple, mathematical model that demonstrates the advantage of a higher reproductive output underlying the JC hypothesis. Finally, we discuss the JC hypothesis in the context of our results and of extinction risk studies on recent animals.

## Materials and Methods

### Analysed classes and orders

#### Aves

We focused on avian orders presumed to have less derived reproductive characteristics (e.g. ground breeding and precocial). According to traditional taxonomy, these are the orders Struthioniformes (n = 6; all analyses) and Tinamiformes (n = 6; all analyses), which are united in the subclass Paleognathae [21], [22], and the orders Galliformes (n = 46; all analyses) and Anseriformes (n = 58; all analyses), which are phylogenetically closely related to the Paleognathae [23]–[26]. We restricted our analyses to ground breeding and terrestrial species (n = 116; all analyses) with an average body mass greater than about 600 g, because this is the approximate weight of small ancestral paravian dinosaurs [27]. Given that sauropods, like most dinosaurs, were most probably ground breeders [28]–[32], we excluded cavity and tree breeding bird species, which are likely to differ profoundly in breeding ecology and life history [33], [34]. Furthermore, the majority of analysed avian species usually do not fly during their routine activities (e.g. foraging and feeding) and are herbivorous.

#### Mammals

In our analysis, we included mammalian species (litter size (LS), n = 353; annual offspring number (AON), n = 203) that belong to the orders Rodentia (LS n = 60; AON n = 32), Lagomorpha (LS n = 14; AON n = 12), Artiodactyla (LS n = 144; AON n = 87), Perissodactyla (LS n = 15; AON n = 11), Hyracoidea (LS n = 3; AON data not available), Proboscidea (LS n = 2; AON n = 2), Diprotodontia (LS n = 35; AON data not available) and Primates (LS n = 80; AON n = 59). Due to the small sample size of Hyracoidea and Proboscidea, we excluded these taxa from the regression analyses carried out for orders, but we included available data in the overall mammalian analyses and in the additional statistical analyses (for details to the additional analyses see below, section Statistical Analyses). All species studied were herbivorous and terrestrial. The smallest species had a minimum average body mass of around 600 g.

#### Dinosaurs

Detailed analyses were performed only for the dinosaurian suborder Sauropoda (sauropods), as the JC hypothesis aims at the understanding of the unique gigantism of dinosaurs. However, to enlarge our dataset, we also compared dinosaur clutch sizes with mammalian litter sizes and avian clutch sizes, considering all dinosaurs for which clutch size and body mass data are currently available (Table S3). In particular, assignments of eggs to producers at present exist only for three theropods (*Troodon formosus*, *Oviraptor philoceratops*, *Citipati osmolskae*), two hadrosaurs (*Maiasaura peeblesorum*, lambeosaurine dinosaur), two sauropods (layers of *Megaloolithus patagonicus*, *Megaloolithus siruguei*) and one prosauropod (*Massospondylus*). All analysed dinosaurs were terrestrial and much larger than 600 g.

### Life history traits and data sources

Avian data on body mass and reproductive biology were collected from the literature (Table S1). The literature was identified through keyword searches in databases of original publications (Web of Science), internet search engines (Google, Google Scholar), as well as individual scanning of references in books and in original publications. Data sets for mammals (Table S2) were exclusively compiled from the database AnAge (Build 10, release date: April 18, 2008) provided by the Human Ageing Genomic Resources project [35]. This database has a good representation of mammalian orders that meet the relevant criteria of our study (body mass >0.600 kg, herbivorous, terrestrial). Information on mammalian diet was taken from Macdonald [36]. For dinosaurs, we collected all data sets that we could find in the literature (Table S3).

We gathered data on body mass, clutch/litter size, breeding frequency, and annual offspring number, for birds and mammals. Annual offspring number was calculated as clutch/litter size multiplied by the number of clutches/litters per year. For body mass we preferred estimates of the mass of females, because mass is more strongly linked to reproductive traits in females than in males. In many cases, however, it was not possible to distinguish between male and female body masses because only averages of both sexes were available. To maximize our sample size while minimizing any bias introduced by male body masses, we used female body masses wherever possible and averaged body masses otherwise. Relative to the range of body sizes included in our analysis (up to 100 kg in birds and up to 4.8 tons in mammals), errors in the estimation of body mass for single species are likely to be negligible.

For dinosaurs, the fossil record provides data on non-sex-specific body mass and clutch size, but not on breeding frequency and annual fecundity.

### Statistical Analyses

To test the assumptions of the JC hypothesis we checked for relationships between body mass and reproductive variables (clutch size and annual offspring number) for birds and mammals using the following statistical methods.

#### Correlation and regression analysis

Augmenting the methods of Janis and Carrano [16], we controlled for phylogenetic dependency of data points [37]–[39]. This is important because body mass is not phylogenetically independent within birds and mammals [40]–[45]. We applied a phylogenetic comparative method (PCM) to control for phylogenetic effects in body mass and reproductive traits. PCMs are generally used to infer to what extent shared traits between species are attributable to common ancestry [38]. However, they are based on the assumption that the traits of interest have evolved in a particular way along a specified phylogenetic tree. Because the underlying evolutionary model could be violated and/or the phylogenetic tree utilized by PCMs could be inaccurate (phylogeny, branch lengths), we performed additional standard analyses based on subsets of species with different body size classes by comparing their means.

#### Phylogenetic comparative methods

As a phylogenetic comparative method, we chose the phylogenetic generalised least square regression (PGLS) [46]–[48], because this method performs well even if the assumptions of a specific evolutionary model are not exactly fulfilled [38], [39]. In general, PCMs perform best when the phylogeny itself and branch lengths are correct [38], [49], [50]. Felsenstein's independent contrasts (PIC) [49] an alternative widely used and well tested phylogenetic method [42], [51] and regression analyses without phylogenetic correction were also conducted. Both statistical methods, however, revealed similar results on the JC hypothesis (Table S4, S5 and S6)

We reconstructed phylogenetic relationships based on published consensus trees derived from morphological and genetic markers when available. To solve the problem that no complete phylogeny was available for all bird species, we constructed a new tree based only on the hierarchies of different published phylogenetic trees of different taxonomic levels (Figure S1). For example, if one phylogeny resolved to genus level and another phylogeny resolved from genus to species level, we fused the two trees. All branch lengths were set to one (with the exception of polytomies, in this case branch lengths were set to 0.0000001), because markers and clustering methods used to construct trees might have been different and thus might have affected branch lengths. If more than one phylogenetic tree was available for one taxonomic level, we chose the best supported one (e.g., different papers and/or methods that result in a similar phylogeny).

For mammals, we used the complete phylogeny given in Bininda-Emonds et al. [52] (tree mammalST_bestDates), excluded all taxa that were not in our mammalian dataset, and set all branch lengths of this tree to one (with the exception of polytomies, in which case branch lengths were again set to 0.0000001) to allow a comparison of results to those obtained for birds (the inclusion of original branch lengths, however, did not change our conclusions on the JC hypothesis).

All correlations and regressions were computed with COMPARE, version 4.6b [53]. We performed significance tests (t-tests) for correlations and differences between slopes of the regression lines obtained for mammals and birds, and for correlations obtained for different avian and mammalian orders. Those tests were calculated using the statistical software R (version 2.7.1). Because the correlations of birds and mammals differ, we only present and discuss these results here. Nevertheless, regression equations obtained for mammals and birds are given in Table S6.

#### Additional analyses on subsets of birds and mammals

We compared the medians of clutch/litter sizes of the orders in each phylogenetic class (birds, mammals) using Kruskal-Wallis tests. Additionally, we grouped birds and mammals into five weight classes. Each class had an equal width of 0.444 on a logarithmic scale. The first class starts at about 0.56 kg (smallest bird), and the last ends at about 93 kg (largest bird). To check for patterns in the average values of reproductive traits, we computed pairwise Wilcoxon tests for birds and mammals of equal weight classes. These tests were also calculated in R.

#### Comparisons between dinosaurs and birds and between dinosaurs and mammals

To compare dinosaur clutch sizes with bird and mammal clutch/litter sizes, we plotted dinosaur data alongside bird and mammal data. In addition, we calculated 95% confidence intervals (prediction intervals) of the regression lines of birds and mammals, respectively.

### Mathematical Model

To demonstrate the advantage of a higher reproductive output underlying the JC hypothesis, we assumed two hypothetical (large) species M and D. The reproductive strategy of species M is comparable to large mammals (with one or two offspring), whereas the strategy of species D is more ratite-like (e.g., ostrich with ten offspring) or dinosaur-like, respectively (megaloolithid clutch size ∼9–25 eggs, [28], [54]–[56]). Both species are iteroparous. Their populations are at equilibrium. Adult mortality is constant and low, and does not differ between species. Births in the population are thus mainly balanced by juvenile mortality (N_off_*S_off_ = 1↔S_off_ = 1/N_off_), where N_off_ is the constant species-specific number of offspring produced per time unit by an adult individual; S_off_ is the survival rate of a juvenile individual and M_off_ = (1-S_off_) its mortality rate. The populations are also large enough that demographic population extinction can be neglected. Species M produces few, large, and well cared offspring (e.g. N_off_ = 1) that have a high survival rate (e.g. S_off_ = 1/N_off_ = 1). Species D has many smaller offspring (e.g. N_off_ = 10) that have a lower survival rate (e.g. S_off_ = 1/N_off_ = 0.1). We suppose that both strategies are equally successful, in terms of that both species have the same number of surviving offspring per time unit (N_off_*S_off_ = 1).

To assess the influence of different juvenile mortalities M_off_ on the number of surviving offspring, we calculated as a conservative approach for species M (N_off_ = 2) and D (N_off_ = 10) the resulting number of surviving offspring considering different juvenile mortalities ranging between 0 and 1 from N_off_*S_off_. This variation in mortalities reflects the impacts of changes in population size on subsequent population growth. To rate the influence of different potential reproductive outputs on the number of surviving offspring we analogously calculated this number for different N_off_ values for species M (N_off_ = 1, S_off_ = 1; N_off_ = 2, S_off_ = 0.5) and species D (N_off_ = 10, S_off_ = 0.1; N_off_ = 20, S_off_ = 0.05). Different N_off_ values mimic species that differ in their reproductive output.

## Results

### Correlation analysis for birds and mammals

#### Clutch/litter size and body mass

We found no correlation between clutch size and body mass in birds (Table 1); neither did we find such a correlation within single bird orders (Table 2).

**Table 1:** Correlations between body mass and reproductive characteristics for birds and mammals.

Correlations	Class	PGLS	N
Body mass vs. clutch/litter size	Birds	0	116
	Mammals	−***	353
Body mass vs. annual offspring number	Birds	+*	116
	Mammals	−***	203

Significance levels: *<0.05, **<0.01, ***<0.001. Correlations are given for double log-transformed data using phylogenetic generalised least square regression (PGLS). 0 no correlation, +significant positive correlation, - significant negative correlation. N sample size.

**Table 2:** Correlations between body mass and reproductive characteristics for different avian and mammalian orders.

	BM vs. clutch/litter size		BM vs. annual offspring number
Order	PGLS	N	PGLS	N
Struthioniformes	0	6	0	6
Tinamiformes	0	6	0	6
Galliformes	0	46	0	46
Anseriformes	0	58	0	58
Rodentia	0	60	0	32
Lagomorpha	0	14	-**	12
Artiodactyla	-*	144	-***	87
Perissodactyla	0	15	-***	11
Primates	0	80	-***	59
Diprotodontia	0	35		

Significance levels:

*<0.05,

**<0.01,

***<0.001.

Correlations are given for double log-transformed data using phylogenetic generalised least square regression (PGLS). 0 no correlation, +significant positive correlation, - significant negative correlation. BM body mass. N sample size.

We found a significant negative correlation between litter size and body mass in mammals (Table 1). This pattern, however, was not found at the level of single orders. We found a significant negative correlation between body mass and litter size in artiodactyls, but none in any of the other mammalian orders (Table 2). The dataset of Hyracoidea and Proboscidea was too small (n<6) to carry out the respective correlation analyses.

#### Annual offspring number and body mass

Birds showed a significant positive correlation between annual offspring number and body mass (Table 1). At the level of single bird orders, we obtained similar results for the relationship between body mass and annual offspring number as seen for body mass versus clutch size (Table 2), because most species have only one brood per year (except for ratites with one to two clutches per year). At this phylogenetic level, we found no correlation between body mass and annual offspring number.

Mammals, however, showed a significant negative correlation between annual offspring number and body mass (Table 1). With one exception (Rodentia), this general pattern was also found within single mammalian orders (Table 2). Rodents showed no correlations between these traits. The dataset of Hyracoidea, Diprotodontia and Proboscidea was too small (n<6) to carry out the respective correlation analyses.

### Additional analyses on subsets of birds and mammals

**Figure 1:**
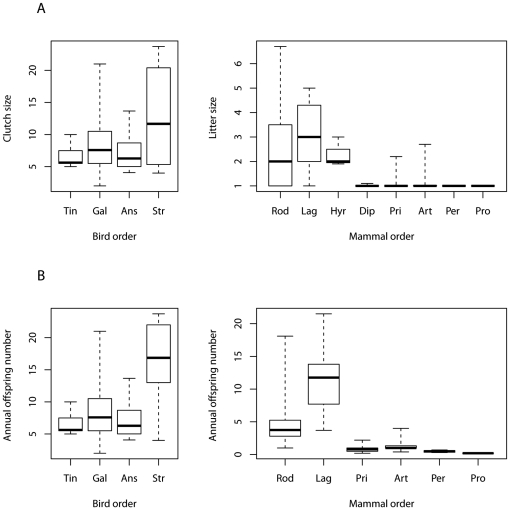
Comparison of the potential reproductive output of bird orders and mammal orders. Potential reproductive output is measured as median clutch/litter size (A) and median annual offspring number (B). Bird orders: Tin = Tinamiformes, Gal = Galliformes, Ans = Anseriformes, Str = Struthioniformes. Mammal orders: Rod = Rodentia, Lag = Lagomorpha, Hyr = Hyracoidea, Dip = Diprotodontia, Pri = Primates, Art = Artiodactyla, Per = Perissodactyla, Pro = Proboscidea. Species orders are ordered by body mass, starting with the lowest median body mass on the left side. All studied bird orders do not differ in their median clutch size, whereas the medians of litter size of studied mammalian orders are inhomogeneous. In birds, the medians of the annual offspring number of all orders are homogeneous, except for the ratites. The medians of annual offspring number of all mammalian orders are inhomogeneous. For the detailed results of the statistical analyses, refer to the text. Box plots show medians, quartiles, minima and maxima of clutch sizes/annual offspring number.

None of the studied bird orders differed in their median clutch size (Kruskal Wallis test: χ^2^ = 5.49, *df* = 3, *p* = 0.139; Figure 1A), whereas the medians of litter size of studied mammalian orders were inhomogeneous (Kruskal Wallis test: χ^2^ = 149.45, *df* = 7, *p*<10^−6^; Figure 1A). In birds, the medians of the annual offspring number of all orders were homogeneous (Kruskal Wallis χ^2^ = 4.04, *df* = 2, *p* = 0.132), except for the ratites, whose median differed from that of the other bird orders (Kruskal Wallis test: χ^2^ = 10.58, *df* = 3, *p* = 0.014; Figure 1B). However, the medians of annual offspring number of all mammalian orders were inhomogeneous (Kruskal Wallis test: χ^2^ = 133.36, *df* = 5, *p*<10^−6^; Figure 1B).

**Figure 2:**
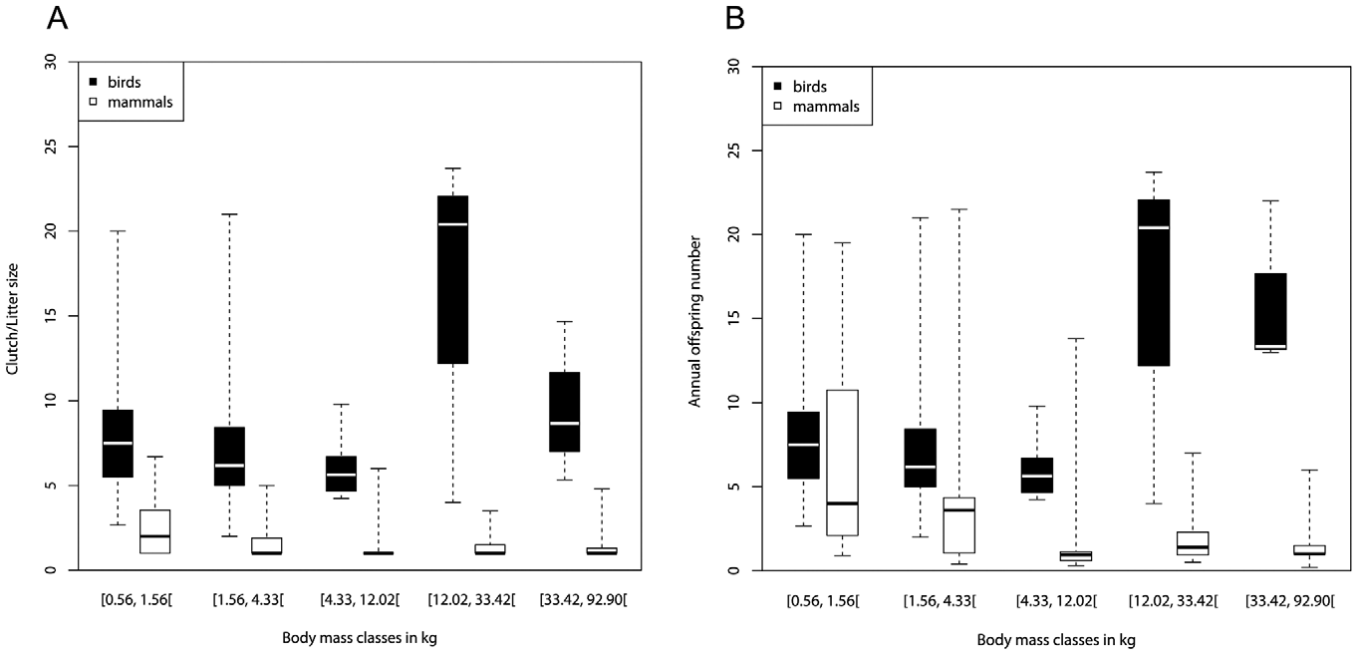
Comparison of the potential reproductive output of birds and mammals for different weight classes. Potential reproductive output is measured as medians of clutch/litter sizes (A) and medians of annual offspring number (B) for species groups of different weight classes. Each weight class interval has a width of 0.444 (unit is kg) on a logarithmic scale. Birds of all weight classes had a higher median clutch/litter size than similar-sized mammals. Similar-sized birds had on average (median) more offspring per year than mammals in all weight classes. For the detailed results of the statistical analyses, refer to the text. Box plots show medians, quartiles, minima and maxima of clutch sizes and litter sizes.

Similar-sized birds of all weight classes had a higher median clutch/litter size than mammals (Figure 2A; exact Wilcoxon tests, N1 = sample size birds, N2 = mammals: Class [0.56, 1.56[, Z = 7.79, N1 = 57, N2 = 39, *p*<10^−6^; Class [1.56, 4.33[, Z = 8.49, N1 = 43, N2 = 57, *p*<10^−6^; Class [4.33, 12.02[, Z = 6.69, N1 = 10, N2 = 87, *p*<10^−6^; Class [12.02, 33.42[, Z = 3.26, N1 = 3, N2 = 43, *p*<10^−4^; Class [33.42, 92.90[, Z = 3.26, N1 = 3, N2 = 58, p<10^−4^) and these differences between birds and mammals tended to be larger in the higher weight classes than in the lower classes (Figure 2A). Similar-sized birds had also on average (median) a higher annual offspring number than mammals in all weight classes (Figure 2B; exact Wilcoxon tests, N1 = sample size birds, N2 = mammals: Class [0.56, 1.56[, Z = 2.28, N1 = 57, N2 = 23, *p* = 0.02; Class [1.56, 4.33[, Z = 4.07, N1 = 43, N2 = 23, *p*<10^−4^; Class [4.33, 12.02[, Z = 4.57, N1 = 10, N2 = 48, *p*<10^−6^; Class [12.02, 33.42[, Z = 2.58, N1 = 3, N2 = 23, *p* = 0.003; Class [33.42, 92.90[, Z = 2.91, N1 = 3, N2 = 33, *p* = 0.0003) and the differences between birds and mammals tended to be larger in the higher weight classes than in the lower ones (Figure 2B).

### Comparisons between dinosaurs and birds and between dinosaurs and mammals

**Figure 3:**
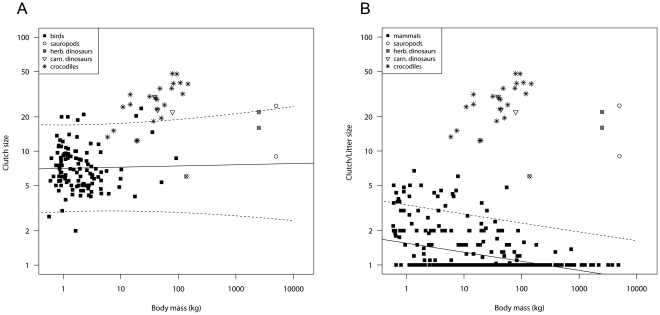
Relationship between clutch/litter size and body mass. (A) in birds, (B) in mammals. Presented are least square regressions (solid lines) and the corresponding 95% confidence intervals (dashed lines, in B only the upper limit of the confidence interval is drawn). Sauropod clutch sizes (open circles) fit well to those of birds or lay somewhat above the upper limit of the confidence interval (A), but do not fit to litter sizes of mammals (B). Carnivorous (open triangles) and other herbivorous dinosaurs (circles with crosses) also fit better to clutch sizes of birds (A) than to litter sizes of mammals (B). Clutch sizes and body masses of dinosaurs are summarized in Table S3. For comparison, clutch sizes of crocodiles were also included (stars). Clutch sizes and body masses of crocodiles are derived from [70].

In general, dinosaur clutch sizes differed from litter sizes of similar-sized mammals but were similar to those of similar-sized birds (Figure 3). Especially sauropod and other herbivorous dinosaur clutch sizes were bird-like (Figure 3A) rather than mammal-like (Figure 3B).

### Mathematical Model

**Figure 4:**
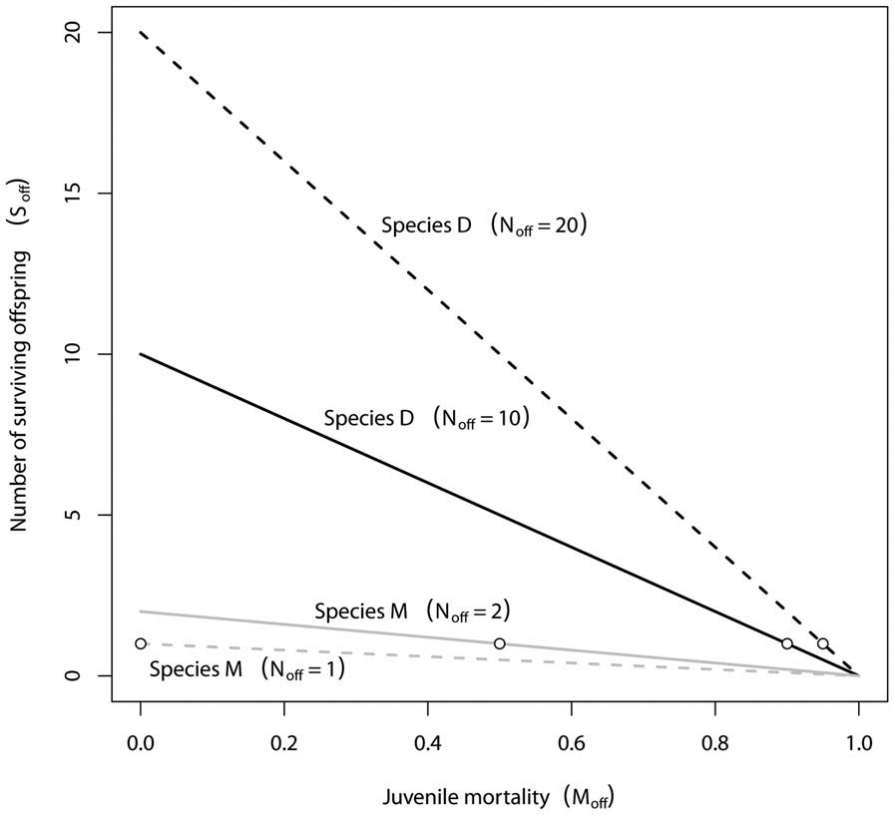
Numbers of surviving offspring (S_off_) of species M (mammal-like) and D (dinosaur-like). S_off_ is shown for different potential reproductive outputs (N_off_) and different juvenile mortalities (M_off_). Species D with the high reproductive output (N_off_ = 10, black solid line or N_off_ = 20, black scattered line) reacts more sensitively to changes in the juvenile mortality rate M_off_ in terms of the number of surviving offspring (S_off_) than species M with the lower reproductive output (N_off_ = 1, grey scattered line or N_off_ = 2, grey solid line). The slopes for M_off_ against S_off_ are much shallower for species M than for species D. The circles mark the (M_off_,S_off_) pairs for which S_off_ equals one surviving offspring (for details see text).

Species D with the high reproductive output (N_off_) reacted more sensitively to changes in the juvenile mortality rate M_off_ than species M with the lower reproductive output (Figure 4). The slope for juvenile mortality against the number of surviving offspring per time unit was much shallower for species M with a low reproductive output than for species D with a high reproductive output (Figure 4).

## Discussion

### Comparison of birds and mammals at class level

Our results corroborated the hypothesis of Janis and Carrano [16] for terrestrial, herbivorous birds and mammals. This hypothesis states that different reproductive strategies have resulted in a different ability of dinosaurs and mammals to evolve and sustain large-bodied species over evolutionary time. Because of their higher potential reproductive output (reflected in clutch/litter size and annual offspring number) when compared to similar-sized mammals, large dinosaurs may have faced a lower risk of extinction under ecological changes. Our analyses revealed that the differences in the life history of birds and mammals proposed by Janis and Carrano [16] exist, such that herbivorous, terrestrial mammals, but not birds, show a significant decrease in clutch/litter size and annual offspring number with increasing body mass.

Our results are supported by Paul's [32], [57] statistical analysis of annual offspring number and body size in reptiles, birds, monotremes, marsupials and placentals. He stated that in extant taxa with an adult mass of 1 g to 10 kg, annual offspring number is broadly similar in egg layers and live bearers. Above 10 kg the number of young of the two types diverges significantly, with many oviparous taxa being much more prolific than mammals. Furthermore, we have shown that birds of all weight classes had a higher median clutch size and annual offspring number than similar-sized mammals. Moreover, these differences between birds and mammals tended to be larger in the higher weight classes than in the lower classes. One reason for this effect could be that, the offspring of large mammals tend to be much bigger than the eggs of large birds. Large mammals have one to two young per year, whereas small mammals, such as rodents, are frequently quite fecund [58], (Figure 1). This bimodal distribution of litter size leads to an overall decrease in litter size and annual offspring number with increasing body size for mammalian vertebrates. In birds, we did not find such a bimodal distribution (Figure 1).

### Comparison of birds and mammals at the level of single orders

The JC hypothesis was also supported by our results when birds and mammals were compared at the level of single orders, except for rodents. In birds, at the level of single orders, and in accordance with the literature [43], [44], we found no correlations using a method that controls for phylogenetic effects (Table 2). In addition, all studied bird orders had similar median clutch sizes and similar annual offspring numbers (with the exception of the ratites). Thus, the potential reproductive output in the different avian orders is very similar (Figure 1).

Conversely, the studied mammalian orders were inhomogeneous in their potential reproductive output and adult body size. Most species from the orders Rodentia and Lagomorpha produce larger litters, have higher annual offspring numbers, and are smaller than species from the orders Artiodactyla, Perissodactyla, Primates, and Diprotodontia (Figure 1). This inhomogeneous distribution in reproductive variables is most probable caused by different development modes. Altricial mammalian species have more and smaller offspring than precocial species [59]. Species from the orders Rodentia and Lagomorpha are mostly altricial, whereas species from the other orders are mainly precocial [59], [60] (primates are intermediate). Therefore, the orders Rodentia and Lagomorpha have on average more offspring (per litter and per year) than other mammalian orders comprising only precocial species (Artiodactyla, Perissodactyla, Primates, Diprotodontia). However, the development mode is only one reason for the inhomogeneous distribution of litter sizes and annual offspring numbers in mammals. Small precocial species from the order Hyracoidea also have higher litter sizes than large precocial species from other orders (Figure 1A). Within the order Artiodactyla, small species have higher litter sizes than large ones (Table 2), and, within the orders Artiodactyla, Perissodactyla and Primates, small species have higher annual offspring numbers than large ones (Table 2).

The observed absent correlation between litter size and body mass in the orders Perissodactyla, Primates and Diprotodontia, and the weak significant negative correlation in artiodactyls, is more or less trivial, because these species have reached the lower limit for litter size producing only one single offspring at a time. In contrast, the absent correlation between body mass and litter size in rodents is not explainable by reaching the lower limit (Figure 1A). The absent correlation between reproductive output and body mass in rodents could be caused by the two different development modes found within rodents (i.e., altricial and precocial) and/or because we did not include many rodents in our study because most rodents are smaller than 600 g.

In addition, it is important to note that all studied large mammalian and all studied large avian species are precocial. Precocial mammalian species generally have fewer offspring than altricial species [59], [60], whereas the opposite is true in birds. Precocial avian species have on average more offspring than altricial birds [33].

### Reproductive output in dinosaurs

The comparison of sauropod clutch sizes to the clutch/litter sizes of hypothetical similar-sized avian or mammalian species demonstrated that dinosaur reproductive output is bird-like (rather than mammal-like). However, this is not to say that reproduction of sauropods resembled reproduction of ancient terrestrial, precocial, herbivorous birds. Additionally, the extrapolation of the bird model to body sizes that are magnitudes larger than those of extant animals could be very erroneous. Furthermore, the data points for clutch size and body mass of sauropods are much less accurate than for birds and mammals. Nevertheless, our results show that the reproductive output of large herbivorous terrestrial mammals is very different from sauropods and, because many species characteristics are shared between birds and dinosaurs [61]–[63], it is probable that some dinosaurs were bird-like in aspects of their reproductive biology. However, we do not know how many clutches sauropods (and dinosaurs in general) had per reproductive event or per breeding season. Sander et al. [28] argue that sauropods might have laid more than one clutch per reproductive event. Evidence for their assumption comes from the large size of adult sauropods in comparison to their small eggs, and the fact that the clutch size of some sauropods might have been limited by physiological constrains of the clutch [55], [64]. If sauropods laid more than one clutch per reproductive event, the “bird model” derived here is wrong, because sauropods would have had a higher reproductive output than recent birds. Results from Grellet-Tinner et al. [65] also call the bird model for sauropods into question. These authors noted that the spatial arrangement of eggs in titanosaur (sauropod) nests [30] and the random spatial distribution of clutches resemble the reproductive mode of modern crocodilians and chelonians, concluding that the titanosaur reproductive mode was probably closer to basal reptilians than to modern birds. In this case, either the true clutch size of sauropods would have resembled extant reptiles, which show an increase in clutch size with increasing body mass (Figure 3, [4], [66]–[70]), or clutch size could alternatively have represented an intermediate state between reptiles and birds. Similar-sized crocodiles and carnivorous dinosaurs have similar clutch sizes, whereas large herbivorous dinosaurs have lower clutch sizes than similar-sized individuals of these two taxa (Figure 3). When the crocodile model applies to the reproductive output of sauropods this implies that large herbivorous dinosaurs must have had several clutches per reproductive event. However, irrespective of whether the reproductive biology of large sauropods was bird-like, reptile-like or intermediate, these animals would always have had a higher potential reproductive output than similar-sized mammals.

### Mathematical model, implications for mammals and dinosaurs

Our simple mathematic model demonstrated that species D reacts more strongly in terms of the number of surviving offspring to changes in juvenile mortality than species M. In the context of the JC-Hypothesis, this observation implies that species D with the higher reproductive output will benefit more strongly from reduced juvenile mortalities resulting from a stochastic reduction in population size than species M. Increased juvenile mortalities, however, are more harmful to species D than to species M. For example, an increase in juvenile mortality M_off_ by 0.01 leads to 0.99 offspring surviving per time unit for species M (N_off_ = 1), whereas for species D (N_off_ = 10) this increase ended up in 0.90 surviving offspring. By contrast, a decrease in M_off_ by 0.01 generated an opposite pattern, with 1.01 offspring surviving for species M (N_off_ = 1) and 1.10 offspring surviving for species D (N_off_ = 10). Thus, when juvenile mortality varies, e.g., as a consequence of a stochastic reduction or increase in population size, species M will suffer less in terms of the number of the surviving offspring from increasing mortalities than species D and will benefit less from decreasing mortalities than species D, whereas species D will suffer more strongly from increased mortalities than species M and will more strongly benefit from decreasing mortalities than species M. Furthermore, this implies that the assumed very high juvenile mortality of species D is rather a maximum value or threshold which should not be often exceeded, whereas the low juvenile mortality in species M is a minimum value.

How applicable are our modelling results on the validity of the JC hypothesis to mammals and in particular to large dinosaurs, e.g., sauropods? Our simple model assumes that all life-cycle characteristics of species M and D are identical, except for their potential reproductive output and their juvenile mortality. Predation and competition for resources, however, are the main agents of mortality in natural populations and may differ between mammals and sauropods. While we can expect that small-bodied sauropod species and small juveniles of sauropods might have experienced a strong predation pressure (and thus high mortality rates as assumed in our model), the predation pressure on large-bodied adult sauropods was probably low and similar to the pressure observed on modern megaherbivores [54]. In addition, age at sexual maturity was estimated to be at one third of the life of a sauropod, and a sauropod's life lasted probably several decades [71]. Thus, these species traits might be comparable in large mammals and sauropods. Such observations suggest that large sauropods could have had adult mortalities similar to those seen in large mammals, as assumed by our model. Nevertheless, evolutionary studies of more complex (realistic) ecological population models are needed to verify the JC-Hypothesis.

### Correlations between reproduction, extinction risk and body size observed in recent animals

Janis and Carrano [16] argue that the difference in the reproductive biologies of dinosaurs and mammals made the dinosaurian clade less vulnerable to extinction. Several studies have shown that the extinction risk of species often correlates with a low reproductive output [72]–[74]. In mammals, large species with a lower reproductive output are at higher risk of extinction than smaller ones [73], [75]. Johnson [74] found the same relationship for the Pleistocene mammalian extinctions and Cardillo [76] for extant terrestrial Australian mammals. In addition, the evidence of the late Pleistocene extinctions illustrates the vulnerability of large mammals to environmental change [77]. Using a large fossil dataset of mammals, Liow et al. [78] described the recurring pattern that large mammal genera and species have higher speciation and extinction rates and therefore existed over shorter times than small ones. Furthermore, the authors found that the differences in extinction rates between large and small mammals are greater than for speciation rates. One explanation for this observation could be that, as Cardillo et al. [73] noted, smaller species are more likely to become threatened simply through environmental risks, whereas for larger species, intrinsic biological traits are an additional significant determinant of extinction risk. This might support the JC hypothesis: an intrinsic trait which could become a significant determinant in large species could be the reproductive output.

However, many species in danger of extinction are large-bodied, a characteristic that leads to low population densities [79], [80]. Low population densities in general lead to high population extinction risk caused by stochastic perturbations independent of the underlying processes (e.g., demographic, environmental, genetic stochasticity, natural catastrophes) even in an environment that is on average favourable for their growth and persistence [80]. Because reproductive output and body size are correlated in mammals, it is difficult to definitely demonstrate if the species' reproductive output, the low population densities or both together imply the higher extinction risk in large mammals than in small ones.

### Reproductive output and gigantism

If a high potential reproductive output enables gigantism, then, as noted by Farlow et al. [81], this raises the question why there were no multi-ton ground birds in the Tertiary. Since the evolution of life history traits is always subject to constraints, other factors may have prevented birds and other taxa with high reproductive output from becoming multi-ton animals. These may be any ecological, morphological or physiological factors in general, or, in the case of the Tertiary birds, competition from mammals, their habit of locomotion, or because they incubate their eggs by body heat. Flightless birds evolved from birds able to fly, which definitely influenced the former's bauplan. In contrast, large mammals evolved from terrestrial animals. Furthermore, as Deeming and Birchard [82] noted, body size is limited in contact incubating birds because of the strength of the eggshell. According to their argument, one would expect that contact incubation will be seen only in small (<250 kg) non-avian theropods [82]. This constraint also could explain that we only find relative gigantism in flightless birds, for example, the moa from New Zealand, the elephant birds from Madagascar or the flightless birds in the Paleogene of the northern hemisphere and throughout the Cenozoic of South America [83]–[86].

Reptiles have a high potential reproductive output (Figure 3, [4], [69]). This raises the question why there were no multi-ton reptiles. Again, only relative gigantism is known from extant tortoises inhabiting several islands (genus *Dipsochelys* from the Seychelles, *Chelonoidis nigra* from Galápagos). A reason why we do not have multi-ton reptiles today could be their slow growth pattern. For example, absolute gigantism is known from the fossils of the giant crocodyliform *Sarcosuchus imperator* from the Cretaceous of Africa [87] which probably had the body mass (8 metric tons) of very large theropods. In this crocodyliform, a maximum adult size was achieved after 50 to 60 years by extending the duration of rapid growth [87].

The above examples suggest that a high reproductive output must not necessarily result in absolute gigantism. Nevertheless, based on our results and all our arguments, we suggest that the reproductive strategy could be one intrinsic species trait which had enabled gigantism in dinosaurs but not in terrestrial mammals.

## Supporting Information

Figure S1 **Phylogenetic tree used to control for phylogenetic effects in body size and reproductive parameters of birds.** * indicates polytomies. Phylogenetic tree was established from references [22], [25], [44], [88]–[109].
(TIF)

Table S1 **Average body mass (BM), clutch size (CS), clutches per year (CY) and offspring per year (OY) for the 116 bird species used in this analyses.** Note: For some species, it was not explicit mentioned in the literature that they have only one clutch per year, nor did we found any evidence that they have usually more than one clutch per year. In this case we assumed one clutch per year as a conservative measure (CY marked with *). Data are from references [110]–[124].
(DOC)

Table S2 **Average body mass (BM), litter size (LS), litters per year (LY) and offspring per year (OY) for the 354 mammal species (LS analyses) or 203 mammal species (OY analyses) used in this study.** Note: the species *Sciurus aberti* (marked with *) was not used in the LS regression analyses because it was not found within the phylogenetic tree we used to control for phylogenetic effects, thus in this case sample size was 353. n.a. = not available. The data for mammals were exclusively compiled from the database AnAge (Build 10, release date: April 18, 2008) provided by the Human Ageing Genomic Resources project [35].
(DOC)

Table S3 **Characteristics of dinosaurs used to test the hypothesis of Janis and Carrano (1992).** Data are from references [28], [55], [56], [63], [125]–[127].
(DOC)

Table S4 **Correlations between body mass and reproductive characteristics for birds and mammals.** Significance levels: *<0.05, **<0.01, ***<0.001. Correlations are given for double log-transformed data using Pearson's correlation coefficient (PEARSON) and two phylogenetic methods (PIC = Felsenstein's independent contrasts; PGLS = phylogenetic generalised least square regression). “**0**” no correlation, “**+**” significant positive correlation, “**−**” significant negative correlation. N: number of species.
(DOC)

Table S5 **Correlations between body mass and reproductive characteristics for different avian and mammalian orders.** Significance levels: *<0.05, **<0.01, ***<0.001. Correlations are given for double log-transformed data using Pearson's correlation coefficient (PEARSON) and two phylogenetic methods (PIC = Felsenstein's independent contrasts; PGLS = phylogenetic generalised least square regression). “**0**” no correlation, “**+**” significant positive correlation, “**−**” significant negative correlation. N: number of species.
(DOC)

Table S6 **Comparison of slopes of regression lines of body mass vs. reproductive characteristics for birds and mammals.** Significance levels: *<0.05, **<0.01, ***<0.001. Regressions were calculated for double log-transformed data using LS (linear least square regression) and two phylogenetic methods (PIC = Felsenstein's independent contrasts; PGLS = phylogenetic generalised least square regression). N: number of species. SE: standard error.
(DOC)
