# Supplementary material for: Reproductive Biology and Its Impact on Body Size: Comparative Analysis of Mammalian, Avian and Dinosaurian Reproduction
Source: PLoS One. 2011 Dec 14;6(12):e28442. doi: 10.1371/journal.pone.0028442 (PMC3237437; doi:10.1371/journal.pone.0028442)
Supplement: Table S1 — Average body mass (BM), clutch size (CS), clutches per year (CY) and offspring per year (OY) for the 116 bird species used in this analyses. Note: For some species, it was not explicit mentioned in the literature that they have only one clutch per year, nor did we found any evidence that they have usually more than one clutch per year. In this case we assumed one clutch per year as a conservative measure (CY marked with *). Data are from references [110]–[124]. (DOC) [file pone.0028442.s002.doc]

**Table S1**. **Average body mass (BM), clutch size (CS), clutches per year (CY) and offspring per year (OY) for the 116 bird species used in this analyses.**

| Species | BM (kg) | CS (#) | CY (#) | OY (#) | References |
| --- | --- | --- | --- | --- | --- |
| **Anseriformes** |  |  |  |  |  |
| *Alopochen aegyptiacus* | 1.914 | 8.50 | 1.00 | 8.50 | [110], [111], [112] |
| *Anas platyrhynchos* | 1.114 | 10.74 | 1.00 | 10.74 | [110], [111], [112] |
| *Anas poecilorhyncha* | 1.095 | 8.50 | 1.00 | 8.50 | [110], [111], [112] |
| *Anas rubripes* | 1.170 | 9.30 | 1.00 | 9.30 | [110], [111], [112] |
| *Anas sparsa* | 0.962 | 5.97 | 1.00 | 5.97 | [110], [111], [112] |
| *Anas undulata* | 0.917 | 7.93 | 1.00 | 7.93 | [110], [111], [112] |
| *Anhima cornuta* | 3.100 | 4.40 | 1.00* | 4.40 | [110], [111], [113] |
| *Anser albifrons* | 2.289 | 5.18 | 1.00 | 5.18 | [110], [111], [112] |
| *Anser anser* | 3.200 | 6.18 | 1.00 | 6.18 | [110], [111], [112] |
| *Anser brachyrhynchus* | 2.788 | 4.10 | 1.00 | 4.10 | [110], [111], [112] |
| *Anser cygnoides* | 3.167 | 5.90 | 1.00 | 5.90 | [110], [112] |
| *Anser erythropus* | 1.860 | 5.00 | 1.00 | 5.00 | [110], [111], [112] |
| *Anser fabalis* | 3.201 | 5.20 | 1.00 | 5.20 | [110], [111], [112] |
| *Anser indicus* | 2.467 | 5.00 | 1.00 | 5.00 | [110], [112] |
| *Anseranas semipalmata* | 2.035 | 8.37 | 1.00 | 8.37 | [110], [112] |
| *Aythya americana* | 1.002 | 9.20 | 1.00 | 9.20 | [110], [111], [112] |
| *Aythya australis* | 0.841 | 11.20 | 1.00 | 11.20 | [110], [111], [112] |
| *Aythya ferina* | 0.913 | 8.77 | 1.00 | 8.77 | [110], [111], [112] |
| *Aythya valisinera* | 1.199 | 9.07 | 1.00 | 9.07 | [110], [111], [112], [114] |
| *Branta bernical* | 1.456 | 4.58 | 1.00 | 4.58 | [110], [111], [112] |
| *Branta canadensis* | 3.430 | 5.73 | 1.00 | 5.73 | [110], [111], [112] |
| *Branta leucopsis* | 1.726 | 4.90 | 1.00 | 4.90 | [110], [111], [112] |
| *Branta ruficollis* | 1.242 | 5.50 | 1.00 | 5.50 | [110], [111], [112] |
| *Branta sandvicensis* | 1.999 | 4.07 | 1.00 | 4.07 | [110], [111], [112] |
| *Cairina scutulata* | 2.568 | 9.67 | 1.00 | 9.67 | [110], [111], [112] |
| *Cereopsis novaehollandiae* | 4.510 | 4.67 | 1.00 | 4.67 | [110], [111] |
| *Chauna torquata* | 4.600 | 4.25 | 1.00 | 4.25 | [110], [111] |
| *Chen caerulescens* | 2.785 | 5.02 | 1.00 | 5.02 | [110], [111], [112] |
| *Chen canagicus* | 2.718 | 4.70 | 1.00 | 4.70 | [110], [111], [112] |
| *Chen rossii* | 1.447 | 4.16 | 1.00 | 4.16 | [110], [111], [112] |
| *Chloephaga hybrida* | 2.183 | 5.10 | 1.00 | 5.10 | [110], [111], [112] |
| *Chloephaga melanoptera* | 3.090 | 7.33 | 1.00 | 7.33 | [110], [112] |
| *Chloephaga picta* | 2.943 | 6.37 | 1.00 | 6.37 | [110], [111], [112] |
| *Chloephaga poliocephala* | 2.222 | 5.00 | 1.00 | 5.00 | [110], [112] |
| *Chloephaga rubidiceps* | 2.000 | 6.00 | 1.00 | 6.00 | [110], [112] |
| *Coscoroba coscoroba* | 4.050 | 5.93 | 1.00 | 5.93 | [110], [111], [112] |
| *Cyanochen cyanopterus* | 1.520 | 6.90 | 1.00 | 6.90 | [110], [111], [112] |
| *Cygnus atratus* | 5.662 | 6.70 | 1.00 | 6.70 | [110], [111], [112] |
| *Cygnus buccinator* | 9.935 | 5.73 | 1.00 | 5.73 | [110], [111], [112] |
| *Cygnus columbianus* | 6.177 | 4.23 | 1.00 | 4.23 | [110], [111], [112] |
| *Cygnus cygnus* | 9.413 | 4.84 | 1.00 | 4.84 | [110], [111], [112] |
| *Cygnus melanacorypha* | 4.550 | 5.53 | 1.00 | 5.53 | [110], [111], [112] |
| *Cygnus olor* | 10.230 | 6.90 | 1.00 | 6.90 | [110], [111], [112] |
| *Dendrocygna arborea* | 1.150 | 9.33 | 1.00* | 9.33 | [110], [112] |
| *Dendrocygna autumnalis* | 0.777 | 13.67 | 1.00 | 13.67 | [110], [111], [112] |
| *Dendrocygna eytoni* | 0.924 | 11.00 | 1.00 | 11.00 | [110], [112] |
| *Hymenolaimus malacorhynchos* | 0.844 | 5.47 | 1.00 | 5.47 | [110], [111], [112] |
| *Lophonetta specularioides* | 0.933 | 6.50 | 1.00 | 6.50 | [110], [111], [112] |
| *Netta erythrophthalma* | 0.794 | 9.67 | 1.00 | 9.67 | [110], [111], [112] |
| *Netta peposaca* | 1.051 | 9.50 | 1.00 | 9.50 | [110], [111], [112] |
| *Netta rufina* | 1.104 | 9.58 | 1.00 | 9.58 | [110], [111], [112] |
| *Oxyura australis* | 0.870 | 5.50 | 1.00 | 5.50 | [110], [111], [112] |
| *Plectropterus gambensis* | 4.480 | 9.80 | 1.00 | 9.80 | [110], [111], [112] |
| *Stictonetta naevosa* | 0.855 | 7.35 | 1.00 | 7.35 | [110], [112] |
| *Tachyeres brachypterus* | 3.680 | 7.00 | 1.00 | 7.00 | [110], [111], [112] |
| *Tachyeres patachonicus* | 2.576 | 6.80 | 1.00 | 6.80 | [110], [111], [112] |
| *Tachyeres pteneres* | 4.566 | 6.53 | 1.00 | 6.53 | [110], [111], [112] |
| *Tadorna ferruginea* | 1.201 | 8.70 | 1.00 | 8.70 | [110], [111], [112] |
|  |  |  |  |  |  |
| **Galliformes** |  |  |  |  |  |
| *Acryllium vulturinum* | 1.269 | 14.00 | 1.00 | 14.00 | [111], [115] |
| *Aepypodius arfakianus* | 1.200 | 20.00 | 1.00 | 20.00 | [115] |
| *Alectura lathami* | 2.270 | 21.00 | 1.00 | 21.00 | [111], [115] |
| *Argusianus argus* | 1.639 | 2.00 | 1.00* | 2.00 | [111], [115] |
| *Catreus wallichii* | 1.218 | 10.50 | 1.00* | 10.50 | [111], [115] |
| *Centrocercus urophasianus* | 1.615 | 10.00 | 1.00 | 10.00 | [111], [115] |
| *Crossoptilon autrium* | 1.665 | 8.67 | 1.00* | 8.67 | [115] |
| *Crossoptilon crossoptilon* | 1.725 | 6.50 | 1.00* | 6.50 | [115] |
| *Crossoptilon mantchuricum* | 1.738 | 13.33 | 1.00* | 13.33 | [115] |
| *Dendragapus obscurus* | 0.865 | 7.00 | 1.00 | 7.00 | [111], [115] |
| *Francolinus castaneicollis* | 0.600 | 5.50 | 1.00* | 5.50 | [115] |
| *Francolinus erckelii* | 1.330 | 7.00 | 1.00* | 7.00 | [111], [115] |
| *Gallus gallus* | 0.678 | 6.00 | 1.00* | 6.00 | [111], [115] |
| *Gallus lafayetii* | 0.567 | 2.67 | 1.00* | 2.67 | [115] |
| *Gallus sonneratii* | 0.748 | 5.00 | 1.00* | 5.00 | [115] |
| *Gallus varius* | 0.623 | 8.67 | 1.00* | 8.67 | [111], [115] |
| *Guttera pucherani* | 1.147 | 5.33 | 1.00 | 5.33 | [111], [115] |
| *Leipoa ocellata* | 1.785 | 18.75 | 1.00 | 18.75 | [115] |
| *Lophophorus impejanus* | 2.033 | 4.50 | 1.00* | 4.50 | [111], [115] |
| *Lophophorus lhuysii* | 3.008 | 4.00 | 1.00* | 4.00 | [115] |
| *Lophura bulweri* | 0.960 | 3.00 | 1.00 | 3.00 | [111], [115] |
| *Lophura diardi* | 0.847 | 6.00 | 1.00 | 6.00 | [111], [115] |
| *Lophura erythrophthalma* | 0.837 | 4.50 | 1.00* | 4.50 | [115] |
| *Lophura leucomelanos* | 0.794 | 7.50 | 1.00* | 7.50 | [115] |
| *Lophura nycthemera* | 1.227 | 9.45 | 1.00* | 9.45 | [111], [115] |
| *Lophura swinhoii* | 1.100 | 6.17 | 1.00* | 6.17 | [115] |
| *Macrocephalon maleo* | 1.650 | 10.00 | 1.00 | 10.00 | [115], [116], [117] |
| *Megapodius nicobariensis* | 0.936 | 20.00 | 1.00 | 20.00 | [115] |
| *Megapodius reinwardt* | 0.881 | 12.50 | 1.00 | 12.50 | [115] |
| *Meleagris gallopavo* | 4.111 | 11.50 | 1.00 | 11.50 | [111], [115] |
| *Meleagris ocellata* | 3.000 | 11.67 | 1.00 | 11.67 | [115] |
| *Numida meleagris* | 1.350 | 9.00 | 1.00 | 9.00 | [111], [115] |
| *Pavo muticus* | 2.230 | 4.50 | 1.00* | 4.50 | [111], [115], [118] |
| *Phasianus colchicus* | 0.984 | 12.17 | 1.00* | 12.17 | [111], [115] |
| *Phasianus versicolor* | 0.831 | 11.00 | 1.00* | 11.00 | [115] |
| *Syrmaticus ellioti* | 0.898 | 6.50 | 1.00* | 6.50 | [111], [115] |
| *Syrmaticus humiae* | 0.750 | 7.50 | 1.00* | 7.50 | [115] |
| *Syrmaticus reevesii* | 0.949 | 7.50 | 1.00* | 7.50 | [115] |
| *Syrmaticus soemmerringii* | 0.907 | 8.67 | 1.00* | 8.67 | [115] |
| *Tetrao parvirostris* | 1.950 | 7.00 | 1.00 | 7.00 | [115] |
| *Tetrao tetrix* | 0.920 | 8.33 | 1.00 | 8.33 | [111], [115] |
| *Tetrao urogallus* | 1.833 | 7.75 | 1.00 | 7.75 | [111], [115] |
| *Tetraogallus altaicus* | 2.540 | 8.20 | 1.00* | 8.20 | [111], [115] |
| *Tetraogallus himalayensis* | 2.686 | 7.67 | 1.00 | 7.67 | [111], [115] |
| *Tetraogallus tibetanus* | 1.385 | 5.00 | 1.00* | 5.00 | [111], [115] |
| *Tragopan satyra* | 1.100 | 3.75 | 1.00* | 3.75 | [115] |
|  |  |  |  |  |  |
| **Struthioniformes** |  |  |  |  |  |
| *Casuarius bennetti* | 17.600 | 4.00 | 1.00* | 4.00 | [110] |
| *Casuarius casuarius* | 51.000 | 5.33 | 2.50 | 13.33 | [110], [111], [119] |
| *Dromaius novaehollandiae* | 35.400 | 14.67 | 1.50 | 22.00 | [110], [111] |
| *Pterocnemia pennata* | 18.500 | 20.40 | 1.00 | 20.40 | [110], [121], [120] |
| *Rhea americana* | 22.667 | 23.71 | 1.00 | 23.71 | [110], [111], [122], [123], [114] |
| *Struthio camelus* | 91.750 | 8.67 | 1.50 | 13.00 | [110], [111], [124] |
|  |  |  |  |  |  |
| **Tinamiformes** |  |  |  |  |  |
| *Nothocerus bonapartei* | 0.871 | 7.50 | 1.00* | 7.50 | [110], [111] |
| *Rhynchotus rufescens* | 0.920 | 5.00 | 1.00 | 5.00 | [110] |
| *Tinamotis pentlandii* | 0.895 | 5.50 | 1.00 | 5.50 | [110] |
| *Tinamus major* | 1.081 | 5.75 | 1.00 | 5.75 | [110], [111] |
| *Tinamus solitarius* | 1.677 | 10.00 | 1.00 | 10.00 | [110], [111] |
| *Tinamus tao* | 1.837 | 5.50 | 1.00 | 5.50 | [110], [111] |

Note: For some species, it was not explicit mentioned in the literature that they have only one clutch per year, nor did we found any evidence that they have usually more than one clutch per year. In this case we assumed one clutch per year as a conservative measure (CY marked with *).
