# Supplementary material for: Reproductive Biology and Its Impact on Body Size: Comparative Analysis of Mammalian, Avian and Dinosaurian Reproduction
Source: PLoS One. 2011 Dec 14;6(12):e28442. doi: 10.1371/journal.pone.0028442 (PMC3237437; doi:10.1371/journal.pone.0028442)
Supplement: Table S2 — Average body mass (BM), litter size (LS), litters per year (LY) and offspring per year (OY) for the 354 mammal species (LS analyses) or 203 mammal species (OY analyses) used in this study. Note: the species Sciurus aberti (marked with *) was not used in the LS regression analyses because it was not found within the phylogenetic tree we used to control for phylogenetic effects, thus in this case sample size was 353. n.a. = not available. The data for mammals were exclusively compiled from the database AnAge (Build 10, release date: April 18, 2008) provided by the Human Ageing Genomic Resources project [35]. (DOC) [file pone.0028442.s003.doc]

**Table S2**. **Average body mass (BM), litter size (LS), litters per year (LY) and offspring per year (OY) for the 354 mammal species (LS analyses) or 203 mammal species (OY analyses) used in this study.**

| Species | BM (kg) | LS (#) | LY (#) | OY (#) |
| --- | --- | --- | --- | --- |
| **Artiodactyla** |  |  |  |  |
| *Addax nasomaculatus* | 92.500 | 1.00 | 1.00 | 1.00 |
| *Aepyceros melampus* | 52.500 | 1.00 | 1.00 | 1.00 |
| *Alcelaphus buselaphus* | 159.00 | 1.00 | 1.00 | 1.00 |
| *Alces alces* | 386.000 | 1.30 | 1.00 | 1.30 |
| *Ammodorcas clarkei* | 30.500 | 1.00 | n.a. | n.a. |
| *Ammotragus lervia* | 92.500 | 1.20 | 2.00 | 2.40 |
| *Antidorcas marsupialis* | 39.000 | 1.00 | 1.00 | 1.00 |
| *Antilocapra americana* | 46.100 | 2.00 | 1.00 | 2.00 |
| *Antilope cervicapra* | 37.500 | 1.00 | 1.90 | 1.90 |
| *Axis axis* | 36.000 | 1.03 | n.a. | n.a. |
| *Axis porcinus* | 43.000 | 1.00 | n.a. | n.a. |
| *Bison bison* | 630.000 | 1.00 | 1.00 | 1.00 |
| *Bison bonasus* | 610.000 | 1.00 | 1.00 | 1.00 |
| *Blastocerus dichotomus* | 102.500 | 1.00 | 1.00 | 1.00 |
| *Bos frontalis* | 825.000 | 1.00 | 0.90 | 0.90 |
| *Bos grunniens* | 667.000 | 1.00 | 0.80 | 0.80 |
| *Bos javanicus* | 700.000 | 1.00 | 1.00 | 1.00 |
| *Bos sauveli* | 800.000 | 1.00 | n.a. | n.a. |
| *Bos taurus* | 750.000 | 1.00 | n.a. | n.a. |
| *Boselaphus tragocamelus* | 180.000 | 1.50 | 1.00 | 1.50 |
| *Bubalus bubalis* | 725.000 | 1.38 | 1.00 | 1.38 |
| *Bubalus depressicornis* | 155.000 | 1.00 | n.a. | n.a. |
| *Bubalus mindorensis* | 240.000 | 1.00 | 0.50 | 0.50 |
| *Budorcas taxicolor* | 180.000 | 1.00 | 1.00 | 1.00 |
| *Camelus bactrianus* | 475.000 | 1.00 | 0.50 | 0.50 |
| *Camelus dromedarius* | 434.000 | 1.00 | 0.70 | 0.70 |
| *Capra caucasica* | 70.000 | 1.00 | n.a. | n.a. |
| *Capra cylindricornis* | 50.000 | 1.20 | 1.00 | 1.20 |
| *Capra falconeri* | 41.000 | 2.00 | n.a. | n.a. |
| *Capra hircus* | 61.000 | 1.50 | 1.00 | 1.50 |
| *Capra ibex* | 82.500 | 1.10 | 0.80 | 0.88 |
| *Capra pyrenaica* | 57.500 | 1.50 | n.a. | n.a. |
| *Capreolus capreolus* | 21.667 | 1.60 | 1.00 | 1.60 |
| *Capreolus pygargus* | 39.450 | 2.00 | 1.00 | 2.00 |
| *Catagonus wagneri* | 34.750 | 2.40 | n.a. | n.a. |
| *Cephalophus dorsalis* | 11.600 | 1.00 | n.a. | n.a. |
| *Cephalophus maxwellii* | 7.000 | 1.00 | 1.00 | 1.00 |
| *Cephalophus monticola* | 6.250 | 1.00 | n.a. | n.a. |
| *Cephalophus natalensis* | 10.000 | 1.00 | n.a. | n.a. |
| *Cephalophus rufilatus* | 13.000 | 1.00 | n.a. | n.a. |
| *Cephalophus silvicultor* | 62.000 | 1.00 | 1.00 | 1.00 |
| *Cephalophus zebra* | 17.500 | 1.00 | n.a. | n.a. |
| *Cervus albirostris* | 135.000 | 1.00 | 1.00 | 1.00 |
| *Cervus duvaucelii* | 170.000 | 1.00 | n.a. | n.a. |
| *Cervus elaphus* | 200.000 | 1.00 | 0.90 | 0.90 |
| *Cervus eldii* | 122.500 | 1.00 | 1.00 | 1.00 |
| *Cervus nippon* | 42.000 | 1.00 | 1.00 | 1.00 |
| *Cervus timorensis* | 63.000 | 1.00 | 1.00 | 1.00 |
| *Cervus unicolor* | 185.000 | 1.00 | 1.00 | 1.00 |
| *Connochaetes gnou* | 132.250 | 1.00 | n.a. | n.a. |
| *Connochaetes taurinus* | 164.500 | 1.00 | 1.00 | 1.00 |
| *Dama dama* | 70.000 | 1.00 | 1.00 | 1.00 |
| *Damaliscus hunteri* | 159.000 | 1.00 | n.a. | n.a. |
| *Damaliscus lunatus* | 110.000 | 1.00 | 0.60 | 0.60 |
| *Damaliscus pygargus* | 84.500 | 1.20 | n.a. | n.a. |
| *Elaphodus cephalophus* | 33.500 | 1.00 | n.a. | n.a. |
| *Elaphurus davidianus* | 186.500 | 1.00 | 1.00 | 1.00 |
| *Gazella cuvieri* | 17.500 | 1.50 | n.a. | n.a. |
| *Gazella dama* | 73.000 | 1.00 | n.a. | n.a. |
| *Gazella dorcas* | 14.500 | 1.00 | 1.00 | 1.00 |
| *Gazella gazella* | 23.250 | 1.00 | 1.50 | 1.50 |
| *Gazella granti* | 67.000 | 1.00 | n.a. | n.a. |
| *Gazella leptoceros* | 48.500 | 1.00 | n.a. | n.a. |
| *Gazella rufifrons* | 15.011 | 1.00 | n.a. | n.a. |
| *Gazella soemmerringii* | 48.500 | 1.00 | n.a. | n.a. |
| *Gazella spekei* | 20.000 | 1.00 | n.a. | n.a. |
| *Gazella subgutturosa* | 48.500 | 1.30 | 1.00 | 1.30 |
| *Gazella thomsonii* | 25.000 | 1.00 | 1.50 | 1.50 |
| *Giraffa camelopardalis* | 800.000 | 1.00 | 0.50 | 0.50 |
| *Hemitragus hylocrius* | 75.000 | 1.30 | n.a. | n.a. |
| *Hemitragus jemlahicus* | 35.200 | 1.00 | 1.00 | 1.00 |
| *Hexaprotodon liberiensis* | 215.000 | 1.00 | 0.40 | 0.40 |
| *Hippocamelus antisensis* | 45.000 | 1.00 | n.a. | n.a. |
| *Hippocamelus bisulcus* | 70.000 | 1.00 | n.a. | n.a. |
| *Hippopotamus amphibius* | 3750.000 | 1.00 | 0.60 | 0.60 |
| *Hippotragus equinus* | 225.000 | 1.00 | n.a. | n.a. |
| *Hippotragus niger* | 225.000 | 1.00 | 1.00 | 1.00 |
| *Hydropotes inermis* | 11.500 | 2.70 | n.a. | n.a. |
| *Hyemoschus aquaticus* | 11.000 | 1.25 | 1.00 | 1.25 |
| *Kobus ellipsiprymnus* | 175.333 | 1.00 | 1.00 | 1.00 |
| *Kobus kob* | 105.000 | 1.00 | 1.30 | 1.30 |
| *Kobus leche* | 94.800 | 1.00 | n.a. | n.a. |
| *Kobus megaceros* | 90.000 | 1.00 | 1.00 | 1.00 |
| *Lama glama* | 140.000 | 1.00 | n.a. | n.a. |
| *Lama guanicoe* | 127.500 | 1.00 | 0.50 | 0.50 |
| *Lama pacos* | 62.000 | 1.04 | n.a. | n.a. |
| *Litocranius walleri* | 43.500 | 1.00 | n.a. | n.a. |
| *Madoqua guentheri* | 4.550 | 1.00 | 2.00 | 2.00 |
| *Madoqua kirkii* | 5.000 | 1.00 | 2.00 | 2.00 |
| *Mazama americana* | 45.500 | 1.30 | 1.00 | 1.30 |
| *Mazama gouazoupira* | 17.000 | 1.00 | n.a. | n.a. |
| *Moschiola meminna* | 2.450 | 1.50 | n.a. | n.a. |
| *Moschus berezovskii* | 12.000 | 1.00 | n.a. | n.a. |
| *Moschus chrysogaster* | 11.000 | 1.00 | n.a. | n.a. |
| *Moschus moschiferus* | 10.000 | 1.50 | 1.00 | 1.50 |
| *Muntiacus muntjak* | 21.000 | 1.17 | n.a. | n.a. |
| *Muntiacus reevesi* | 18.000 | 1.00 | 1.50 | 1.50 |
| *Naemorhedus crispus* | 80.000 | 1.00 | n.a. | n.a. |
| *Naemorhedus goral* | 31.750 | 1.00 | 1.00 | 1.00 |
| *Naemorhedus sumatraensis* | 95.000 | 1.00 | 1.00 | 1.00 |
| *Neotragus moschatus* | 6.500 | 1.00 | n.a. | n.a. |
| *Odocoileus hemionus* | 57.000 | 1.50 | 1.00 | 1.50 |
| *Odocoileus virginianus* | 87.000 | 2.00 | 1.00 | 2.00 |
| *Okapia johnstoni* | 225.000 | 1.00 | 0.80 | 0.80 |
| *Oreamnos americanus* | 90.000 | 1.20 | 1.00 | 1.20 |
| *Oreotragus oreotragus* | 12.000 | 1.00 | 1.00 | 1.00 |
| *Oryx dammah* | 177.500 | 1.00 | n.a. | n.a. |
| *Oryx gazella* | 170.000 | 1.00 | 1.30 | 1.30 |
| *Oryx leucoryx* | 121.350 | 1.00 | 1.00 | 1.00 |
| *Ourebia ourebi* | 17.000 | 1.00 | 1.50 | 1.50 |
| *Ovibos moschatus* | 315.000 | 1.00 | 0.80 | 0.80 |
| *Ovis ammon* | 160.000 | 1.00 | 1.00 | 1.00 |
| *Ovis aries* | 110.000 | 1.58 | 1.00 | 1.58 |
| *Ovis canadensis* | 70.275 | 1.00 | 1.00 | 1.00 |
| *Ovis dalli* | 73.100 | 1.00 | 1.00 | 1.00 |
| *Ovis vignei* | 60.000 | 1.50 | n.a. | n.a. |
| *Ozotoceros bezoarticus* | 32.500 | 1.00 | 1.00 | 1.00 |
| *Pecari tajacu* | 20.200 | 2.00 | 2.00 | 4.00 |
| *Pelea capreolus* | 25.000 | 1.00 | n.a. | n.a. |
| *Procapra gutturosa* | 30.000 | 1.30 | 1.00 | 1.30 |
| *Pseudois nayaur* | 55.000 | 1.50 | n.a. | n.a. |
| *Pudu puda* | 10.000 | 1.00 | n.a. | n.a. |
| *Rangifer tarandus* | 101.250 | 1.00 | 1.00 | 1.00 |
| *Raphicerus campestris* | 13.000 | 1.00 | n.a. | n.a. |
| *Redunca arundinum* | 58.000 | 1.00 | n.a. | n.a. |
| *Redunca fulvorufula* | 30.000 | 1.00 | 1.10 | 1.10 |
| *Redunca redunca* | 45.500 | 1.00 | n.a. | n.a. |
| *Rupicapra rupicapra* | 37.500 | 1.00 | 1.00 | 1.00 |
| *Saiga tatarica* | 37.500 | 1.70 | 1.00 | 1.70 |
| *Sylvicapra grimmia* | 18.500 | 1.00 | 1.40 | 1.40 |
| *Syncerus caffer* | 700.000 | 1.00 | 0.40 | 0.40 |
| *Taurotragus derbianus* | 680.000 | 1.00 | n.a. | n.a. |
| *Taurotragus oryx* | 500.000 | 1.00 | n.a. | n.a. |
| *Tayassu pecari* | 22.000 | 2.00 | n.a. | n.a. |
| *Tetracerus quadricornis* | 19.000 | 1.50 | n.a. | n.a. |
| *Tragelaphus angasii* | 120.000 | 1.00 | 1.20 | 1.20 |
| *Tragelaphus eurycerus* | 307.500 | 1.00 | 0.70 | 0.70 |
| *Tragelaphus imberbis* | 82.500 | 1.00 | 1.30 | 1.30 |
| *Tragelaphus scriptus* | 60.000 | 1.00 | 1.50 | 1.50 |
| *Tragelaphus spekii* | 87.500 | 1.00 | 1.00 | 1.00 |
| *Tragelaphus strepsiceros* | 217.500 | 1.00 | 1.00 | 1.00 |
| *Tragulus javanicus* | 3.850 | 1.00 | n.a. | n.a. |
| *Tragulus napu* | 6.500 | 1.00 | n.a. | n.a. |
| *Vicugna vicugna* | 50.000 | 1.00 | 0.70 | 0.70 |
|  |  |  |  |  |
| **Diprotodontia** |  |  |  |  |
| *Dendrolagus bennettianus* | 9.300 | 1.00 | n.a. | n.a. |
| *Dendrolagus dorianus* | 9.581 | 1.00 | n.a. | n.a. |
| *Dendrolagus goodfellowi* | 7.400 | 1.00 | n.a. | n.a. |
| *Dendrolagus inustus* | 11.400 | 1.00 | n.a. | n.a. |
| *Dendrolagus matschiei* | 7.200 | 1.00 | n.a. | n.a. |
| *Dendrolagus ursinus* | 8.000 | 1.00 | n.a. | n.a. |
| *Dorcopsis luctuosa* | 3.570 | 1.00 | n.a. | n.a. |
| *Dorcopsis muelleri* | 5.000 | 1.00 | n.a. | n.a. |
| *Lagorchestes conspicillatus* | 3.000 | 1.00 | n.a. | n.a. |
| *Lagorchestes hirsutus* | 1.310 | 1.00 | n.a. | n.a. |
| *Lagostrophus fasciatus* | 2.100 | 1.00 | n.a. | n.a. |
| *Macropus agilis* | 16.000 | 1.00 | n.a. | n.a. |
| *Macropus antilopinus* | 49.500 | 1.00 | n.a. | n.a. |
| *Macropus dorsalis* | 11.250 | 1.00 | n.a. | n.a. |
| *Macropus eugenii* | 6.500 | 1.00 | n.a. | n.a. |
| *Macropus fuliginosus* | 28.250 | 1.00 | n.a. | n.a. |
| *Macropus giganteus* | 49.500 | 1.00 | n.a. | n.a. |
| *Macropus parma* | 4.250 | 1.00 | n.a. | n.a. |
| *Macropus parryi* | 16.500 | 1.00 | n.a. | n.a. |
| *Macropus robustus* | 30.000 | 1.00 | n.a. | n.a. |
| *Macropus rufogriseus* | 16.200 | 1.00 | n.a. | n.a. |
| *Macropus rufus* | 55.000 | 1.00 | n.a. | n.a. |
| *Onychogalea fraenata* | 5.500 | 1.00 | n.a. | n.a. |
| *Onychogalea unguifera* | 5.800 | 1.00 | n.a. | n.a. |
| *Petrogale assimilis* | 4.300 | 1.00 | n.a. | n.a. |
| *Petrogale brachyotis* | 3.700 | 1.00 | n.a. | n.a. |
| *Petrogale concinna* | 1.250 | 1.00 | n.a. | n.a. |
| *Petrogale lateralis* | 4.200 | 1.00 | n.a. | n.a. |
| *Petrogale penicillata* | 6.000 | 1.00 | n.a. | n.a. |
| *Petrogale xanthopus* | 6.000 | 1.10 | n.a. | n.a. |
| *Setonix brachyurus* | 3.500 | 1.00 | n.a. | n.a. |
| *Thylogale billardierii* | 5.000 | 1.00 | n.a. | n.a. |
| *Thylogale stigmatica* | 4.100 | 1.00 | n.a. | n.a. |
| *Thylogale thetis* | 3.800 | 1.00 | n.a. | n.a. |
| *Wallabia bicolor* | 14.625 | 1.00 | n.a. | n.a. |
|  |  |  |  |  |
| **Hyracoidea** |  |  |  |  |
| *Dendrohyrax arboreus* | 3.000 | 2.00 | n.a. | n.a. |
| *Heterohyrax brucei* | 2.457 | 1.90 | n.a. | n.a. |
| *Procavia capensis* | 3.600 | 3.00 | n.a. | n.a. |
|  |  |  |  |  |
| **Lagomorpha** |  |  |  |  |
| *Bunolagus monticularis* | 1.250 | 1.00 | n.a. | n.a. |
| *Lepus americanus* | 1.600 | 3.00 | 2.60 | 7.80 |
| *Lepus californicus* | 4.175 | 1.00 | 3.70 | 3.70 |
| *Lepus capensis* | 2.358 | 2.60 | 3.30 | 8.58 |
| *Lepus europaeus* | 4.175 | 2.00 | 3.80 | 7.60 |
| *Lepus timidus* | 4.175 | 2.00 | 2.10 | 4.20 |
| *Lepus townsendii* | 3.036 | 4.30 | 2.80 | 12.04 |
| *Oryctolagus cuniculus* | 1.800 | 5.00 | 4.30 | 21.50 |
| *Sylvilagus audubonii* | 0.900 | 3.00 | 5.00 | 15.00 |
| *Sylvilagus bachmani* | 0.700 | 3.60 | 3.20 | 11.52 |
| *Sylvilagus floridanus* | 1.150 | 5.00 | 3.90 | 19.50 |
| *Sylvilagus nuttallii* | 0.760 | 3.60 | 3.50 | 12.60 |
| *Sylvilagus palustris* | 1.233 | 2.30 | n.a. | n.a. |
| *Sylvilagus transitionalis* | 0.903 | 4.80 | 2.50 | 12.00 |
|  |  |  |  |  |
| **Perissodactyla** |  |  |  |  |
| *Ceratotherium simum* | 2180.000 | 1.00 | 0.40 | 0.40 |
| *Dicerorhinus sumatrensis* | 1260.000 | 1.00 | 0.30 | 0.30 |
| *Diceros bicornis* | 1100.000 | 1.00 | 0.40 | 0.40 |
| *Equus asinus* | 164.998 | 1.00 | n.a. | n.a. |
| *Equus burchellii* | 280.000 | 1.00 | 0.60 | 0.60 |
| *Equus caballus* | 250.000 | 1.00 | n.a. | n.a. |
| *Equus grevyi* | 384.000 | 1.00 | 0.50 | 0.50 |
| *Equus hemionus* | 230.000 | 1.00 | 0.70 | 0.70 |
| *Equus zebra* | 296.000 | 1.00 | 0.60 | 0.60 |
| *Rhinoceros sondaicus* | 1750.000 | 1.00 | 0.30 | 0.30 |
| *Rhinoceros unicornis* | 1600.000 | 1.00 | 0.30 | 0.30 |
| *Tapirus bairdii* | 300.000 | 1.00 | 0.70 | 0.70 |
| *Tapirus indicus* | 250.000 | 1.00 | 0.50 | 0.50 |
| *Tapirus pinchaque* | 148.950 | 1.00 | n.a. | n.a. |
| *Tapirus terrestris* | 250.000 | 1.00 | n.a. | n.a. |
|  |  |  |  |  |
| **Primates** |  |  |  |  |
| *Allenopithecus nigroviridis* | 4.703 | 1.00 | n.a. | n.a. |
| *Cacajao calvus* | 3.165 | 1.00 | 0.50 | 0.50 |
| *Cacajao melanocephalus* | 2.935 | 1.00 | n.a. | n.a. |
| *Callicebus cupreus* | 1.120 | 1.00 | 0.90 | 0.90 |
| *Callicebus moloch* | 0.804 | 1.00 | 1.00 | 1.00 |
| *Cercocebus galeritus* | 9.468 | 1.00 | n.a. | n.a. |
| *Cercocebus torquatus* | 9.493 | 1.00 | 0.90 | 0.90 |
| *Cercopithecus ascanius* | 3.705 | 1.00 | 0.70 | 0.70 |
| *Cercopithecus campbelli* | 3.600 | 1.00 | n.a. | n.a. |
| *Cercopithecus cephus* | 3.585 | 1.00 | 0.40 | 0.40 |
| *Cercopithecus diana* | 4.550 | 1.00 | 1.00 | 1.00 |
| *Cercopithecus lhoesti* | 4.700 | 1.00 | 0.80 | 0.80 |
| *Cercopithecus mitis* | 9.000 | 1.00 | 0.50 | 0.50 |
| *Cercopithecus mona* | 4.500 | 1.00 | n.a. | n.a. |
| *Cercopithecus neglectus* | 5.945 | 1.00 | 1.00 | 1.00 |
| *Cercopithecus nictitans* | 5.465 | 1.00 | 0.50 | 0.50 |
| *Cercopithecus pogonias* | 3.580 | 1.00 | 0.50 | 0.50 |
| *Cercopithecus solatus* | 5.405 | 1.00 | 0.70 | 0.70 |
| *Chiropotes albinasus* | 2.910 | 1.00 | n.a. | n.a. |
| *Chiropotes satanas* | 2.943 | 1.00 | n.a. | n.a. |
| *Chlorocebus aethiops* | 5.620 | 1.00 | 1.00 | 1.00 |
| *Colobus angolensis* | 8.625 | 1.00 | n.a. | n.a. |
| *Colobus guereza* | 9.950 | 1.00 | 1.00 | 1.00 |
| *Colobus polykomos* | 9.525 | 1.00 | 0.60 | 0.60 |
| *Erythrocebus patas* | 7.750 | 1.00 | 1.00 | 1.00 |
| *Eulemur coronatus* | 2.500 | 1.50 | 1.00 | 1.50 |
| *Eulemur fulvus* | 3.150 | 1.10 | 2.00 | 2.20 |
| *Eulemur macaco* | 2.500 | 1.00 | 1.00 | 1.00 |
| *Eulemur mongoz* | 2.060 | 1.10 | 1.00 | 1.10 |
| *Eulemur rubriventer* | 1.765 | 1.00 | n.a. | n.a. |
| *Gorilla gorilla* | 139.842 | 1.00 | 0.30 | 0.30 |
| *Hapalemur griseus* | 1.348 | 1.20 | 1.10 | 1.32 |
| *Hapalemur simus* | 1.738 | 1.00 | n.a. | n.a. |
| *Hylobates agilis* | 5.925 | 1.00 | 0.30 | 0.30 |
| *Hylobates concolor* | 6.853 | 1.00 | n.a. | n.a. |
| *Hylobates hoolock* | 6.875 | 1.00 | 0.30 | 0.30 |
| *Hylobates klossii* | 5.900 | 1.00 | n.a. | n.a. |
| *Hylobates lar* | 6.810 | 1.00 | 0.50 | 0.50 |
| *Hylobates moloch* | 6.208 | 1.00 | n.a. | n.a. |
| *Hylobates muelleri* | 5.765 | 1.00 | n.a. | n.a. |
| *Hylobates pileatus* | 5.735 | 1.00 | n.a. | n.a. |
| *Hylobates syndactylus* | 10.900 | 1.00 | 0.40 | 0.40 |
| *Lemur catta* | 2.555 | 1.10 | 0.90 | 0.99 |
| *Lophocebus albigena* | 9.318 | 1.00 | 0.40 | 0.40 |
| *Macaca arctoides* | 9.275 | 1.00 | 0.60 | 0.60 |
| *Macaca fascicularis* | 6.363 | 1.00 | 0.90 | 0.90 |
| *Macaca fuscata* | 8.883 | 1.50 | 0.50 | 0.75 |
| *Macaca maura* | 5.575 | 1.00 | 0.60 | 0.60 |
| *Macaca mulatta* | 8.235 | 1.00 | 1.00 | 1.00 |
| *Macaca nemestrina* | 7.913 | 1.00 | 0.80 | 0.80 |
| *Macaca nigra* | 7.965 | 1.00 | 0.70 | 0.70 |
| *Macaca radiata* | 6.755 | 1.00 | 1.00 | 1.00 |
| *Macaca silenus* | 7.875 | 1.00 | 0.70 | 0.70 |
| *Macaca sinica* | 4.370 | 1.00 | 0.70 | 0.70 |
| *Macaca sylvanus* | 10.875 | 1.50 | 1.00 | 1.50 |
| *Macaca thibetana* | 10.300 | 1.00 | 0.50 | 0.50 |
| *Mandrillus leucophaeus* | 18.250 | 1.00 | 0.80 | 0.80 |
| *Mandrillus sphinx* | 23.000 | 1.00 | 0.80 | 0.80 |
| *Miopithecus talapoin* | 1.385 | 1.00 | 1.00 | 1.00 |
| *Nasalis larvatus* | 14.618 | 1.25 | 0.70 | 0.88 |
| *Pan paniscus* | 39.925 | 1.00 | 0.20 | 0.20 |
| *Pan troglodytes* | 44.984 | 1.00 | 0.20 | 0.20 |
| *Papio hamadryas* | 18.000 | 1.00 | 0.80 | 0.80 |
| *Pithecia monachus* | 1.780 | 1.00 | n.a. | n.a. |
| *Pithecia pithecia* | 1.480 | 1.00 | 1.00 | 1.00 |
| *Pongo pygmaeus* | 64.475 | 1.00 | 0.20 | 0.20 |
| *Presbytis comata* | 6.695 | 1.00 | n.a. | n.a. |
| *Presbytis melalophos* | 6.540 | 1.00 | n.a. | n.a. |
| *Propithecus diadema* | 5.550 | 1.00 | 0.50 | 0.50 |
| *Propithecus verreauxi* | 5.000 | 1.00 | 1.00 | 1.00 |
| *Pygathrix nemaeus* | 9.720 | 1.00 | 1.00 | 1.00 |
| *Semnopithecus entellus* | 13.517 | 1.00 | 0.70 | 0.70 |
| *Theropithecus gelada* | 16.200 | 1.00 | 0.50 | 0.50 |
| *Trachypithecus cristatus* | 6.185 | 1.00 | n.a. | n.a. |
| *Trachypithecus geei* | 8.100 | 1.00 | n.a. | n.a. |
| *Trachypithecus johnii* | 12.000 | 1.00 | n.a. | n.a. |
| *Trachypithecus obscurus* | 7.080 | 1.00 | 0.50 | 0.50 |
| *Trachypithecus phayrei* | 8.400 | 1.00 | 0.80 | 0.80 |
| *Trachypithecus vetulus* | 7.082 | 1.00 | 0.40 | 0.40 |
| *Varecia variegata* | 3.670 | 2.20 | 1.00 | 2.20 |
|  |  |  |  |  |
| **Proboscidea** |  |  |  |  |
| *Elephas maximus* | 3180.000 | 1.00 | 0.20 | 0.20 |
| *Loxodonta africana* | 4800.000 | 1.00 | 0.20 | 0.20 |
|  |  |  |  |  |
| **Rodentia** |  |  |  |  |
| *Agouti paca* | 9.000 | 1.00 | 1.50 | 1.50 |
| *Aplodontia rufa* | 1.125 | 2.75 | 1.00 | 2.75 |
| *Atherurus africanus* | 2.750 | 1.50 | 2.50 | 3.75 |
| *Atherurus macrourus* | 2.000 | 1.25 | n.a. | n.a. |
| *Cannomys badius* | 0.650 | 1.80 | n.a. | n.a. |
| *Capromys pilorides* | 4.683 | 2.00 | n.a. | n.a. |
| *Castor canadensis* | 20.250 | 3.50 | 1.00 | 3.50 |
| *Castor fiber* | 25.000 | 3.00 | 1.00 | 3.00 |
| *Cavia porcellus* | 0.728 | 3.80 | n.a. | n.a. |
| *Cavia tschudii* | 0.638 | 1.90 | n.a. | n.a. |
| *Chinchilla lanigera* | 0.643 | 2.00 | 2.00 | 4.00 |
| *Coendou prehensilis* | 3.900 | 1.00 | n.a. | n.a. |
| *Cynomys gunnisoni* | 0.900 | 4.40 | 1.00 | 4.40 |
| *Cynomys ludovicianus* | 1.125 | 4.00 | 1.00 | 4.00 |
| *Dasyprocta cristata* | 2.65 | 2.00 | n.a. | n.a. |
| *Dasyprocta leporina* | 3.265 | 1.00 | n.a. | n.a. |
| *Dasyprocta punctata* | 2.150 | 2.00 | n.a. | n.a. |
| *Dinomys branickii* | 12.250 | 2.00 | n.a. | n.a. |
| *Dolichotis patagonum* | 12.500 | 2.00 | 3.50 | 7.00 |
| *Erethizon dorsatum* | 8.600 | 1.00 | 1.00 | 1.00 |
| *Geocapromys brownii* | 1.500 | 2.00 | 2.00 | 4.00 |
| *Geocapromys ingrahami* | 0.660 | 1.00 | n.a. | n.a. |
| *Hydrochaeris hydrochaeris* | 55.000 | 4.80 | 1.25 | 6.00 |
| *Hypogeomys antimena* | 1.250 | 1.00 | n.a. | n.a. |
| *Hystrix africaeaustralis* | 24.000 | 2.10 | 1.70 | 3.57 |
| *Hystrix brachyura* | 8.000 | 1.50 | n.a. | n.a. |
| *Hystrix cristata* | 20.000 | 2.30 | 2.00 | 4.60 |
| *Hystrix indica* | 20.000 | 2.00 | n.a. | n.a. |
| *Kerodon rupestris* | 0.950 | 1.50 | n.a. | n.a. |
| *Lagidium peruanum* | 1.250 | 1.00 | 2.00 | 2.00 |
| *Lagidium viscacia* | 1.540 | 1.50 | n.a. | n.a. |
| *Lagostomus maximus* | 6.000 | 2.00 | 1.80 | 3.60 |
| *Lophiomys imhausi* | 0.755 | 1.75 | n.a. | n.a. |
| *Marmota bobak* | 7.300 | 4.50 | 0.50 | 2.25 |
| *Marmota caligata* | 6.343 | 4.20 | 0.50 | 2.10 |
| *Marmota flaviventris* | 3.500 | 4.00 | 0.90 | 3.60 |
| *Marmota marmota* | 3.500 | 4.00 | 1.00 | 4.00 |
| *Marmota monax* | 4.000 | 4.50 | 1.00 | 4.50 |
| *Marmota vancouverensis* | 4.750 | 3.30 | 0.50 | 1.65 |
| *Mesembriomys gouldii* | 0.900 | 2.00 | 4.00 | 8.00 |
| *Myocastor coypus* | 7.850 | 6.00 | 2.30 | 13.80 |
| *Myoprocta acouchy* | 0.775 | 2.00 | n.a. | n.a. |
| *Ondatra zibethicus* | 1.362 | 6.70 | 2.70 | 18.09 |
| *Pedetes capensis* | 3.500 | 1.00 | 3.60 | 3.60 |
| *Petaurista magnificus* | 1.780 | 1.00 | n.a. | n.a. |
| *Petaurista petaurista* | 1.750 | 1.00 | n.a. | n.a. |
| *Petaurista philippensis* | 1.652 | 1.00 | n.a. | n.a. |
| *Phloeomys cumingi* | 1.750 | 1.00 | n.a. | n.a. |
| *Plagiodontia aedium* | 1.267 | 1.00 | n.a. | n.a. |
| *Ratufa bicolor* | 2.000 | 1.40 | 2.00 | 2.80 |
| *Ratufa indica* | 2.000 | 1.00 | n.a. | n.a. |
| *Sciurus aberti** | 0.703 | 3.50 | 1.50 | 5.25 |
| *Sciurus aureogaster* | 0.600 | 2.00 | n.a. | n.a. |
| *Sciurus niger* | 0.800 | 3.00 | 1.00 | 3.00 |
| *Sciurus vulgaris* | 0.600 | 5.00 | 2.00 | 10.00 |
| *Spermophilus variegatus* | 0.663 | 4.30 | 1.50 | 6.45 |
| *Sphiggurus mexicanus* | 2.000 | 1.00 | n.a. | n.a. |
| *Sphiggurus villosus* | 1.750 | 1.00 | n.a. | n.a. |
| *Thryonomys swinderianus* | 6.600 | 4.00 | 2.30 | 9.20 |
| *Xerus erythropus* | 0.600 | 3.50 | n.a. | n.a. |
| *Xerus inauris* | 0.588 | 2.20 | 1.00 | 2.20 |

Note: the species *Sciurus aberti* (marked with *) was not used in the LS regression analyses because it was not found within the phylogenetic tree we used to control for phylogenetic effects, thus in this case sample size was 353. n.a. = not available.

The data for mammals were exclusively compiled from the database AnAge (Build 10, release date: April 18, 2008) provided by the Human Ageing Genomic Resources project [35].
