# Supplementary material for: Reproductive Biology and Its Impact on Body Size: Comparative Analysis of Mammalian, Avian and Dinosaurian Reproduction
Source: PLoS One. 2011 Dec 14;6(12):e28442. doi: 10.1371/journal.pone.0028442 (PMC3237437; doi:10.1371/journal.pone.0028442)
Supplement: Table S3 — Characteristics of dinosaurs used to test the hypothesis of Janis and Carrano (1992). Data are from references [28], [55], [56], [63], [125]–[127]. (DOC) [file pone.0028442.s004.doc]

**Table S3.** **Characteristics of dinosaurs used to test the hypothesis of Janis and Carrano (1992).**

| Species | Classification | Body mass (kg) | Clutch size | Reference(s) |
| --- | --- | --- | --- | --- |
| *Troodoon formosus* | Carnivore | 43.700 | 23 | [63] |
| *Oviraptor philoceratops* | Carnivore | 39.000 | 30 | [63] |
| *Citipati osmolskae* | Carnivore | 79.000 | 22 | [63] |
| *Maiasaura peeblesorum* | Herbivore | 2500.000 | 16 | [63],[125] |
| lambeosaurine | Herbivore | 2500.000 | 22 | [63],[125] |
| *Massospondylus* | Herbivore | 137.000 | 6 | [126],[127] |
| *Megaloolithus patagonicus* | Sauropod | 5000.000 | 25 | [63],[55],[28] |
| *Megaloolithus sirguei* | Sauropod | 5000.000 | 9 | [63],[55],[28], |
| *Megaloolithus sirguei* | Sauropod | 5000.000 | 25 | [63],[56] |
