# Supplementary material for: Reproductive Biology and Its Impact on Body Size: Comparative Analysis of Mammalian, Avian and Dinosaurian Reproduction
Source: PLoS One. 2011 Dec 14;6(12):e28442. doi: 10.1371/journal.pone.0028442 (PMC3237437; doi:10.1371/journal.pone.0028442)
Supplement: Table S4 — Correlations between body mass and reproductive characteristics for birds and mammals. Significance levels: *<0.05, **<0.01, ***<0.001. Correlations are given for double log-transformed data using Pearson's correlation coefficient (PEARSON) and two phylogenetic methods (PIC = Felsenstein's independent contrasts; PGLS = phylogenetic generalised least square regression). “0” no correlation, “+” significant positive correlation, “−” significant negative correlation. N: number of species. (DOC) [file pone.0028442.s005.doc]

**Table S4.** **Correlations between body mass and reproductive characteristics for birds and mammals.**

| Correlations | Class | PEARSON | PIC | PGLS | N |
| --- | --- | --- | --- | --- | --- |
| Body mass vs. clutch/litter size | Birds | **0** | **0** | **0** | 116 |
|  | Mammals | **-***** | **-***** | **-***** | 353 |
| Body mass vs. annual offspring number | Birds | **0** | **+*** | **+*** | 116 |
|  | Mammals | **-***** | **-***** | **-***** | 203 |

Significance levels: * < 0.05, ** < 0.01, *** < 0.001.

Correlations are given for double log-transformed data using Pearson’s correlation coefficient (PEARSON) and two phylogenetic methods (PIC = Felsenstein’s independent contrasts; PGLS = phylogenetic generalised least square regression). “**0**” no correlation, “**+**“ significant positive correlation, “**-**“ significant negative correlation. N: number of species
