# Supplementary material for: Reproductive Biology and Its Impact on Body Size: Comparative Analysis of Mammalian, Avian and Dinosaurian Reproduction
Source: PLoS One. 2011 Dec 14;6(12):e28442. doi: 10.1371/journal.pone.0028442 (PMC3237437; doi:10.1371/journal.pone.0028442)
Supplement: Table S5 — Correlations between body mass and reproductive characteristics for different avian and mammalian orders. Significance levels: *<0.05, **<0.01, ***<0.001. Correlations are given for double log-transformed data using Pearson's correlation coefficient (PEARSON) and two phylogenetic methods (PIC = Felsenstein's independent contrasts; PGLS = phylogenetic generalised least square regression). “0” no correlation, “+” significant positive correlation, “−” significant negative correlation. N: number of species. (DOC) [file pone.0028442.s006.doc]

**Table S5.** **Correlations between body mass and reproductive characteristics for different avian and mammalian orders.**

| Order | body mass vs. clutch/litter size | | | |  | body mass vs. annual offspring number | | | |
| --- | --- | --- | --- | --- | --- | --- | --- | --- | --- |
|  | PEARSON | PIC | PGLS | N |  | PEARSON | PIC | PGLS | N |
| Struthioniformes | **0** | **0** | **0** | 6 |  | **0** | **0** | **0** | 6 |
| Tinamiformes | **0** | **0** | **0** | 6 |  | **0** | **0** | **0** | 6 |
| Galliformes | **0** | **0** | **0** | 46 |  | **0** | **0** | **0** | 46 |
| Anseriformes | **-***** | **0** | **0** | 58 |  | **-***** | **0** | **0** | 58 |
| Rodentia | **0** | **0** | **0** | 60 |  | **0** | **0** | **0** | 32 |
| Lagomorpha | **0** | **0** | **0** | 14 |  | **-***** | **0** | **-**** | 12 |
| Artiodactyla | **-*** | **-*** | **-*** | 144 |  | **-***** | **-***** | **-***** | 87 |
| Perissodactyla | **0** | **0** | **0** | 15 |  | **-***** | **-*** | **-***** | 11 |
| Primates | **0** | **0** | **0** | 80 |  | **-***** | **-***** | **-***** | 59 |
| Diprotodontia | **0** | **0** | **0** | 35 |  |  |  |  |  |

Significance levels: * < 0.05, ** < 0.01, *** < 0.001.

Correlations are given for double log-transformed data using Pearson’s correlation coefficient (PEARSON) and two phylogenetic methods (PIC = Felsenstein’s independent contrasts; PGLS = phylogenetic generalised least square regression). “**0**” no correlation, “**+**“ significant positive correlation, “**-**“ significant negative correlation. N: number of species.
