# Supplementary material for: Reproductive Biology and Its Impact on Body Size: Comparative Analysis of Mammalian, Avian and Dinosaurian Reproduction
Source: PLoS One. 2011 Dec 14;6(12):e28442. doi: 10.1371/journal.pone.0028442 (PMC3237437; doi:10.1371/journal.pone.0028442)
Supplement: Table S6 — Comparison of slopes of regression lines of body mass vs. reproductive characteristics for birds and mammals. Significance levels: *<0.05, **<0.01, ***<0.001. Regressions were calculated for double log-transformed data using LS (linear least square regression) and two phylogenetic methods (PIC = Felsenstein's independent contrasts; PGLS = phylogenetic generalised least square regression). N: number of species. SE: standard error. (DOC) [file pone.0028442.s007.doc]

**Table S6.** **Comparison of slopes of regression lines of body mass vs. reproductive characteristics for birds and mammals.**

|  | | |  |  | Method | | |
| --- | --- | --- | --- | --- | --- | --- | --- |
|  | | |  |  | LS | PIC | PGLS |
| Body mass vs. clutch/litter size | | | | |  |  |  |
|  | Birds (N = 116): | | | |  |  |  |
|  |  | Intercept (SE) | | | 0.85 (0.02) | 0.76 (0.03) | 0.82 (0.08) |
|  |  | Slope (SE) | | | 0.01 (0.04) | 0.09 (0.06) | 0.06 (0.06) |
|  | Mammals (N = 353): | | | |  |  |  |
|  |  | Intercept (SE) | | | 0.19 (0.02) | -0.21 (0.00) | 0.16 (1.18) |
|  |  | Slope (SE) | | | -0.08 (0.01) | -0.05 (0.02) | -0.05 (0.02) |
|  | Different slopes in birds and mammals | | | | Yes* | Yes* | No |
| Body mass vs. annual offspring number | | | | |  |  |  |
|  | Birds (N = 116): | | | |  |  |  |
|  |  | Intercept (SE) | | | 0.84 (0.02) | 0.78 (0.03) | 0.82 (0.07) |
|  |  | Slope (SE) | | | 0.07 (0.05) | 0.15 (0.06) | 0.12 (0.06) |
|  | Mammals (N = 203): | | | |  |  |  |
|  |  | Intercept (SE) | | | 0.43 (0.04) | 3.11 (0.00) | 2.09 (0.60) |
|  |  | Slope (SE) | | | -0.24 (0.03) | -0.95 (0.00) | -0.95 (0.00) |
|  | Different slopes in birds and mammals | | | | Yes*** | Yes*** | Yes*** |

Significance levels: * < 0.05, ** < 0.01, *** < 0.001.

Regressions were calculated for double log-transformed data using LS (linear least square regression) and two phylogenetic methods (PIC = Felsenstein’s independent contrasts; PGLS = phylogenetic generalised least square regression). N: number of species. SE: standard error.
